# Regulatory Role of Silicon in Mediating Differential Stress Tolerance Responses in Two Contrasting Tomato Genotypes Under Osmotic Stress

**DOI:** 10.3389/fpls.2018.01475

**Published:** 2018-10-08

**Authors:** Nusrat Ali, Adrian Schwarzenberg, Jean-Claude Yvin, Seyed A. Hosseini

**Affiliations:** Plant Nutrition Department, Centre Mondial de l’Innovation Roullier, Saint-Malo, France

**Keywords:** tomato, metabolites, amino acids, polyamines, mineral nutrition and redox state

## Abstract

Previous studies have shown the role of silicon (Si) in mitigating the adverse effect of drought stress in different crop species. However, data are lacking on a comparison of drought tolerant and drought sensitive crop cultivars in response to Si nutrition. Therefore, the aim of this study was to elucidate the mechanism (s) by which two contrasting tomato genotypes respond to Si nutrition under osmotic stress condition. Two tomato lines contrasting in their response to drought stress were hydroponically grown under polyethylene glycol (PEG, 6000) and two regimes of Si (0 and 1.5 mM). Metabolite profiling was performed in two lines. Growth and relevant physiological parameters, and expression levels of selected genes were also measured. Si application resulted in improved osmotic stress tolerance in both drought tolerant line LA0147 and drought sensitive line FERUM. In the drought tolerant line, Si enhanced uptake of sulfur (S) and ammonium (${\text{NH}}_{4}^{+}$) which led to a significantly higher production of amino acids arginine, methionine, serine, and glycine. While in the drought sensitive line, Si significantly increased production of amino acids proline and GABA which further lowered the level of GSSG to GSH ratio and thus balanced the redox homeostasis under osmotic stress. The higher significant production of amino acids arginine, methionine, GABA, and proline enhanced production of free polyamines putrescine and spermidine and improved osmotic stress tolerance. Therefore, we conclude that Si distinctively regulated osmotic stress tolerance in two contrasting tomato genotypes by differential accumulation of relevant amino acids which eventually led to enhanced polyamine metabolism.

## Introduction

Abiotic stress is estimated to reduce the yield of crops by 51–82% (Cooke and Leishman, 2016). Plants are frequently subjected to environmental constraints and water scarcity is one of the most important abiotic stress limiting crop and yield production worldwide. Therefore, plants have evolved different morpho-physiological and biochemical strategies to cope with drought stress (Ijaz et al., 2017). These adaptive strategies are regulated by an intricate signaling network and regulated stress-responsive gene expression (Ramegowda et al., 2014). Despite the internal mechanism (s) which plants employ to cope with drought stress, increasing evidence suggests that the detrimental effects of drought can be mitigated by the adequate and balanced supply of mineral nutrients (Waraich et al., 2011). In particular the possible mechanisms through which potassium, nitrogen, and sulfur nutrition can minimize the detrimental effects of drought in plants has been well investigated (Chan et al., 2013; Wei et al., 2013; Gimeno et al., 2014). Several studies have reported that Silicon (Si) application to crops enhances their tolerance to drought (Gong et al., 2008; Chen et al., 2011; Amin et al., 2016), and so plants use Si as a ‘quasi-essential’ element in order to alleviate the impacts of drought stress (Ma et al., 2004; Pei et al., 2010). In these studies, the beneficial effects of Si were primarily due to its role through increasing anti-oxidant production, maintaining photosynthetic machinery (Shen et al., 2010) and delaying leaf senescence (Hosseini et al., 2017). In a study in tomato, Si mitigated drought-induced oxidative stress by activating antioxidant enzymes and decreasing malondialdehyde concentrations (Shi et al., 2014). Besides, enhanced antioxidant activities, Si has also been shown to enhance radial hydraulic conductivity and mediate stress tolerance in tomato plants exposed to drought (Cao et al., 2017). The above-mentioned studies have assessed the impact of Si to mitigate drought stress only in single species. Despite the importance of Si nutrition in drought tolerance, a comparison of drought tolerant and drought sensitive crop cultivars has not yet been reported. Thus, it has remained an open question, whether supplemental Si is able to counteract drought stress more effectively in drought-tolerant and/or in drought sensitive genotypes.

Plants also undergo a wide range of metabolic changes as end products of cellular regulatory processes in response to drought stress (Zhao et al., 2014). The most common stress tolerance strategies in plants are an accumulation of highly soluble low-molecular weight compounds (Monti et al., 2006). These metabolites function as osmolytes to maintain the structure of cellular components that enable plants to cope better with drought conditions (Kang et al., 2011; Chan et al., 2013). Previous studies have shown that sugars, sugar alcohols, some amino acids, and amines such as proline, glycine, betaine, and polyamines accumulate upon drought stress in different plant species (Seki et al., 2007). The metabolic response to drought stress in varieties with different responses to drought stress has been well studied in crops like rice (Zhao et al., 2014), soybean (Silvente et al., 2012), alfalfa (Kang et al., 2011), and maize (Witt et al., 2012). Changes in the metabolite levels have also been reported in response to different individual mineral deficiencies such as potassium (Armengaud et al., 2009), nitrogen (Nagy et al., 2013), phosphorus and sulfur (Amtmann and Armengaud, 2009). In regard to Si nutrition, so far there is only one research study which indicated that Si modulates carbon/nitrogen balance in unstressed rice plants (Detmann et al., 2012a). These authors highlighted the important role of Si which acts as a signal in promoting amino acids remobilization. They found a positive correlation between Si concentration and the levels of amino acids alanine, arginine, glutamine, ornithine, isoleucine, methionine, and valine. More recent evidence highlighting an effect of Si on plant metabolism comes from a study on *Sorghum* plants under drought stress, where Si induces the accumulation of polyamines (PAs) at the expenses of 1-aminocyclopropane-1-carboxylic acid (ACC) (Yin et al., 2014). However, it is still open which role Si takes in drought-induced metabolite changes and which metabolite contributes more to protect stressed plants treated with Si.

To answer these questions, we performed metabolite profiling in two tomato lines contrasting in their response to drought stress. Plants were grown hydroponically and PEG was used for simulating osmotic stress. After Si treatment, the growth and relevant physiological parameters, as well as elemental analysis were determined. In addition, transcript regulation of the genes involved in the respective metabolite pathways were examined. The results clearly showed that Si resulted in a higher uptake and translocation of both ${\text{NH}}_{4}^{+}$ and S in a drought tolerant line under osmotic stress conditions (OSC). Metabolite profiling also revealed distinct changes in the amino acids arginine (Arg) and methionine (Met), upon Si supply in the drought tolerant line, whereas the sensitive line showed higher levels of proline (Pro) and gamma-aminobutyric acid (GABA). The increase in the level of these amino acids eventually led to an accumulation of polyamines, and so enhancing osmotic stress tolerance.

## Materials and Methods

### Plant Materials and Growth Conditions

Tomato (*Solanum lycopersicum* L.) seeds of both drought tolerant (LA0147) and drought sensitive (FERUM) genotypes were kindly provided by Centre de Recherche PACA, INRA (Avignon, France). Seeds of both genotypes were germinated on moist vermiculite (equilibrated with water) for 3 days in the dark followed by a further 4 days in light conditions. After 1 week, the seedlings of both LA0147 and FERUM were grown hydroponically in the greenhouse and maintained at a day/night temperature of 25°C/22°C and a day/night cycle of 16 h/8 h. Until the two-leaf stage, for each genotype all plants were divided into two groups: -Si/-PEG and Si/-PEG pre-cultured in 0.75 mM monosilicic acid. These plants were grown in complete nutrient solution containing Ca(NO_3_)_2_ 2 mM, K_2_SO_4_ 1 mM, MgSO_4_ 0.5 mM, NH_4_H_2_PO_4_ 0.5 mM, CaCl_2_ 0.5 mM, H_3_BO_3_ 0.001 mM, MnSO_4_ 0.0025 mM, ZnSO_4_ 0.0005 mM, CuSO_4_ 0.0002 mM, (NH_4_)_6_Mo_7_O_24_ 0.00001 mM, and 0.1 mM of EDTA iron (III) sodium salt. The nutrient solution was buffered to pH 5.9 and renewed twice a week with continuous aeration. Monosilicic acid [Si(OH)_4_], was freshly prepared by passing sodium silicate solution through a column filled with cation-exchange resins according to Yin et al. (2013). For each genotype, after emergence of two true leaves, plants were divided into three batches and the osmotic stress was simulated in the second and third batch by addition of 1% PEG (PEG-6000) in the nutrient solution, with supplemental Si (1.5 mM) only for the third batch (Cao et al., 2017). The plants of the first batch were kept under control conditions without supplying PEG and Si. For each treatment, four independent samples of LA0147 and FERUM were harvested 21 days after transplanting and immediately frozen in liquid nitrogen and stored at -80°C until further molecular and biochemical analysis.

### Chlorophyll Measurement

Leaf chlorophyll content was estimated non-destructively in fully expanded leaves (3 plants per treatments) using a portable chlorophyll meter DUALEX- V4 (Force A, Orsay, France).

### Determination of Sulfur Content

For determination of sulfur content, roots and leaves samples were dried for 48 h at 65°C (Multiwave PRO, Anton Paar) and digested using 8 mL of concentrated HNO_3_ (65% Merck). Elements were analyzed by Inductively Coupled Plasma Optical Emission Spectrometry (iCAP 6500 dual OES spectrometer, Thermo Scientific, Waltham, MA, United States) by using Yttrium solution (1 ppm, Merck) as an internal standard.

### Determination of Anions and Cations

Sulfate and ammonium were measured using high-performance liquid chromatography with a conductivity detector (ICS5000+, Thermo Scientific-Dionex, Villebon-sur-Yvette, France). Both sulfate and ammonium were extracted as described in Abdallah et al. (2010). Gradients of potassium hydroxide (KOH) and methane sulfonic acid (MSA) were used to perform the separation between anions and cation over an analytical columns AS19 and CS12, respectively.

### Metabolite Analysis

Metabolite extraction was conducted using 20 mg of frozen ground fresh leaves and roots, which were weighed in a 2 mL Eppendorf tubes, then 500 μL of cold water/methanol 70:30 v/v (-20°C) containing 0.4% of perchloric acid (v/v) solvent was added. Samples were shaken by vortex mixing for 10 min then centrifuged using an Eppendorf Centrifuge 5427 R (Hamburg, Germany) for 15 min at 12,700 RPM at 4°C. Supernatants were collected and introduced into new 2 mL Eppendorf tubes. A second extraction was performed adding 500 μL of water +0.1% perchloric acid (v/v) to leaves and roots, vortex mixed for 5 min, and centrifuged for 15 min at 12,700 RPM at 4°C. Supernatants were mixed and centrifuged for 10 min in order to eliminate suspended particles. Finally, supernatants were diluted two times with water + 0.1% formic acid (v/v) and introduced in 2 mL LC–MS vials.

Metabolite analysis was achieved using an ultra-high performance liquid chromatography (UPLC) Acquity H-Class system (Waters Corp, Milford, MA, United States), and a high resolution detection was performed by using a Xevo G2-S QToF mass spectrometer (Waters Corp, Milford, MA, United States) equipped with an electrospray ionization (ESI) source. Two column chemistries were used in order to retain different metabolites. A Waters UPLC HSS T3 column (2.1 mm × 100 mm, 1.8 μm) was used to profile polar metabolites such as amino acids and sulfur containing metabolites. The mobile phase comprising water containing 0.1% formic acid (A) and acetonitrile: methanol (50:50 v/v) containing 0.1% formic acid (B) was applied with an optimized gradient elution as follows: 100% A at 0–1.5 min, 100–80% A at 1.5–2 min, 80–20% A at 2–2.5 min, 20% A at 2.5–4.5 min, 20–100% A at 4.5–5 min, 100% A at 5–7 min. The flow rate was kept at 0.4 mL/min, and column temperature was maintained at 25°C. The ESI source was used in positive ionization mode, the voltage was set to 0.5 kV and cone voltage was 15 V, and source temperature was maintained at 130°C with a cone gas flow of 20 L/h. Desolvation temperature was at 500°C with desolvation gas flow of 800 L/h. A Phenomenex Luna^®^ Omega PS C18 (100 μm × 2.1 μm, 1.6 μm) column (Torrance, CA, United States) was used to profile metabolites such as organic acids. The mobile phase comprised of water containing 0.5% formic acid (A) and methanol : water (70:30 v/v) containing 0.5% formic acid (B) which was applied with the optimized gradient elution as follows: 100% A at 0–1 min, 100–20% A at 1–4 min, 20–0% A at 4–6.5 min, 0% A at 6.5–7.5 min, 0–100% A at 7.5–7.9 min, 100% A at 7.9–10 min. The flow rate was kept at 0.3 mL/min, column temperature was maintained at 35°C. The injection volume for both columns was 10 μL and samples were maintained at 10°C. The ESI source was used in negative ionization mode, source voltage was set to 2.5 kV and cone voltage was 30 V, whilst source temperature was maintained at 130°C with a cone gas flow of 20 L/h. Desolvation temperature was at 550°C with desolvation gas flow of 900 L/h. Leucine-Enkephalin was used as lockmass reference (ion at m/z 556.2771 in positive mode), which was introduced by a lockspray at 10 μL min^-1^ for real-time data calibration. The MS^E^ data were acquired in centroid mode using a scan range 50–800 Da, scan time 0.1 s, resolution was set at 20000 full width half maximum (FWHM), and a collision energy ramp 40–80 V.

For those metabolites which were not detected, we used the same UPLC Acquity H-Class system, but with different detector (PDA) and column (BEH C18, 100 mm × 2.1 mm, 1.7 μm) for detection and measurement using the AccQ-Tag procedure (Waters, United States). Amino acids derivatization with AccQ-Tag was performed according to the Waters protocol. Briefly, 10 μL of samples and standard were mixed with 70 μL of borate buffer and 20 μL of AccQ-Tag reagent. Then, vials were heated 10 min at 55°C. Chromatographic separation was performed using four mobile phases as Waters recommendations with some optimization: (A) ammonium formate 50 mM/ACN (90:10 v:v) at pH 3.2, (B) H_2_O/ACN 2% formic acid (90:10 v:v), (C) H_2_O, and (D) ACN 2% formic acid. The gradient elution as follows: 10% A/90% B at 0–3.70 min, 9% A/80% B/11% C at 3.70–6 min, 9% A/80% B/11% D at 6.0–10.0 min, 10% A/90% C at 10.0–11.0 min, 10% A/90% C at 11.0–15.0 min. The flow rate was kept at 0.7 mL/min, column temperature was maintained at 48°C. The injection volume for both columns was 1 μL and samples were maintained at 20°C.

Data treatment, alignment, peak picking, normalization, deconvolution, and multivariate analysis was performed using Progenesis QI software (Nonlinear Dynamics, Newcastle, United Kingdom) and EZinfo 3.0 software (Umetrics AB, Umeå, Sweden). Concentration of all metabolites and amino acids were calculated by external calibration based on analytical standards using TargetLynx^TM^ software (Waters, United States) (Ghosson et al., 2018). Statistical analysis was achieved using MetaboAnalyst (Xia et al., 2015).

Polyamine extraction was achieved using 10 mg of frozen ground leaves that were weighed in a 2 mL eppendorf tube (Eppendorf, Germany). Extraction was carried out by adding 1 mL of a solution of 70% H_2_O/29% MeOH/1.0% formic acid (v:v:v) at -20°C. Then, the tubes were stirred at room temperature for 30 min, then centrifuged at 4°C (16000 rpm) and the supernatant was transferred into new Eppendorf tubes. The supernatant was transferred to a LC/MS vial for analysis. Polyamines were analyzed by an UHPLC–MS/MS system. The separation and detection were achieved using a Nexera X2 UHPLC system (Shimadzu, Japan) coupled to a QTrap 6500+ mass spectrometer (Sciex, Canada) equipped with an IonDrive^TM^ turbo V electrospray (ESI) source.

### RNA Extraction and Gene Expression Analysis

Total RNA was extracted from root (100 mg) and leaf samples (70 mg) of tomato plants for each treatments using Nucleospin^®^ 8 RNA kit following the manufacturer’s protocol (Macherey-Nagel, Düren, Germany). RNA quality and quantity were determined using a 4200 Tapestation (Agilent Technologies, Santa Clara, CA, United States), followed by DNase treatment and cDNA synthesis from 1 μg RNA using iScript gDNA clear Kit containing iScript Reverse Transcription Supermix (BIO-RAD, Berkeley, CA, United States). Quantitative real-time PCR (qPCR) analysis was performed in a total volume of 10 μl using Universal SYBR Green Supermix (BIO-RAD, Berkeley, CA, United States) in Real-Time PCR Detection System (BIO-RAD, Berkeley, CA, United States). All the qPCR reactions were performed in technical triplicates using independent cDNA reactions for each biological replicate and 300 nM of gene-specific primer pairs. Specific primers for all candidate genes were designed using Primer3 software and listed in **Supplementary Table S1**. The cycling conditions were as follows: pre-denaturation at 98°C for 3 min, 40 cycles of 98°C for 15 s (denaturation), 60°C for 30 s (annealing) and 72°C for 15 s (extension), a final extension at 72°C for 5 min followed by a melting curve analysis to confirm the correct amplification of target gene fragments. The expression of all candidate genes was normalized against four reference genes, namely *SAND*, *CAC*, *ACTIN*, and *EF1α* (Expósito-Rodríguez et al., 2008). All qPCR expression data were acquired and analyzed using CFX Maestro Software Version 1.0 (BIO-RAD, Berkeley, CA, United States).

### Statistical Analysis

For each genotype, individual treatments on tomato were conducted under control and OSCs (with or without Si), with four independent biological replicates. Data are represented as mean ± standard error (SE) for *n* = 4. Statistical analyses were carried out by one way ANOVA and significant differences were analyzed by Fischer’s LSD method. Data are marked by different letters when significantly different (*p* < 0.05).

## Results

### LA0147 Shows Better Tolerance to Osmotic Stress Compared to FERUM

**FIGURE 1:**
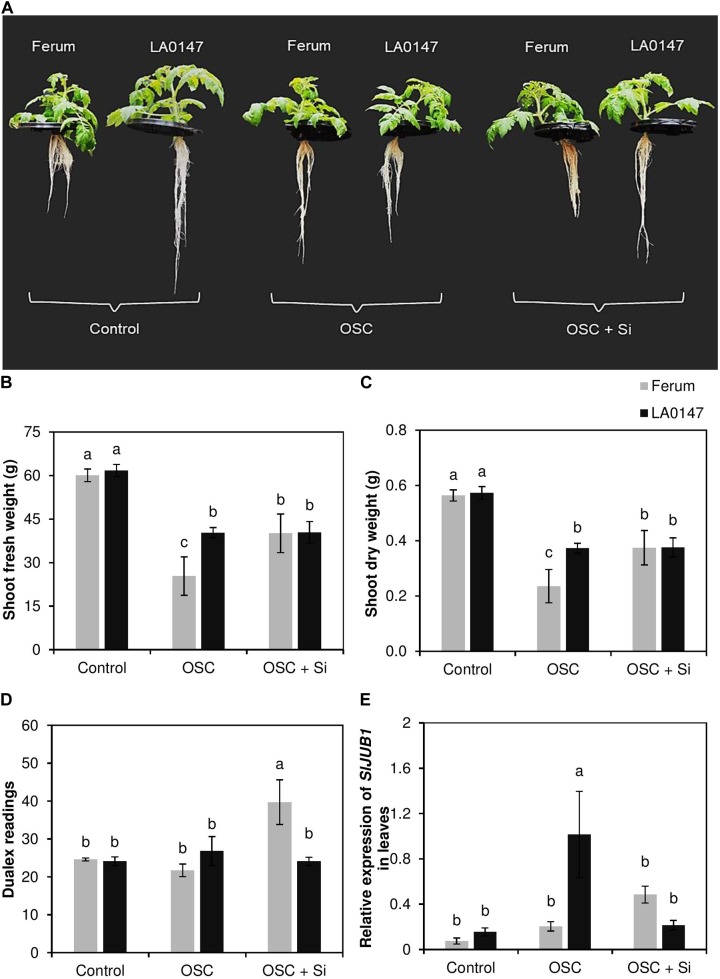
Influence of Si supply on shoot fresh and dry weights, chlorophyll level, and expression pattern of drought marker gene in two contrasting tomato genotypes under osmotic stress. **(A)** Monitoring both root and shoot phenotype under control, OSC and OSC + Si, **(B)** shoot fresh weight, **(C)** shoot dry weight **(D)**, chlorophyll levels and **(E)** relative expression of *SlJUB1*. Plants were grown in hydroponic culture and osmotic stress was simulated by applying polyethylene glycol (PEG 6000). Si was provided at 0.75 mM for pre-cultured plants and at 1.5 mM for osmotic stressed plants. Roots and fully expanded leaves from 21-day old plants were harvested 7 days after imposition of osmotic stress. Bars indicate means ± SE. Different letters denote significant differences according to Fischer’s LSD test (*p* < 0.05; *n* = 4).

To prove the differential responses of LA0147 and FERUM to drought stress, we measured physiological and molecular markers of drought stress in both lines. The relevant physiological parameters revealed that the level of both shoot fresh and dry weights were identical in both lines under control condition (**Figures 1A–C**). Conversely, under OSC, the tolerant line LA0147 showed significantly higher biomass compared to the sensitive line FERUM (**Figures 1B,C**). Application of Si did not change the biomass of tolerant line LA0147 under OSC, whereas the biomass of sensitive line FERUM significantly increased by Si supply under OSC (**Figures 1B,C**). The root growth parameters were also analyzed, however in spite of visual differences in root morphology, no significant changes were observed in either root biomass (fresh and dry weight) or in the root length of both lines in response to different treatments (**Supplementary Figure S1**). In addition, chlorophyll was measured by a dualex device, but no significant differences were observed under control conditions or OSC in either genotypes (**Figure 1D**). Interestingly, the sensitive line FERUM showed a significantly higher dualex value under OSC in Si-treated plants compared to tolerant line LA0147 (**Figure 1D**). We further examined the expression level of the NAC transcription factor *JUNGBRUNNEN1* (*JUB1*) as a regulator of abiotic stress tolerance in tomato (Shahnejat-Bushehri et al., 2016). The expression level of this gene was significantly induced and was almost six times higher in tolerant line LA0147 relative to sensitive line FERUM (**Figure 1E**) indicating that LA0147 is more osmotic stress tolerant compared to sensitive line FERUM. In addition, Si supply suppressed the expression level of *SlJUB1* in tolerant line LA0147 under OSC while it was induced in sensitive line FERUM (**Figure 1E**). These results showed that under OSC the LA0147 coped better with osmotic stress compared to the sensitive line and Si supply increased the growth level of sensitive line FERUM under OSC.

### Genotype Specific Differences Lead to Differential Nutrient Uptake in Response to Si Supply

**FIGURE 2:**
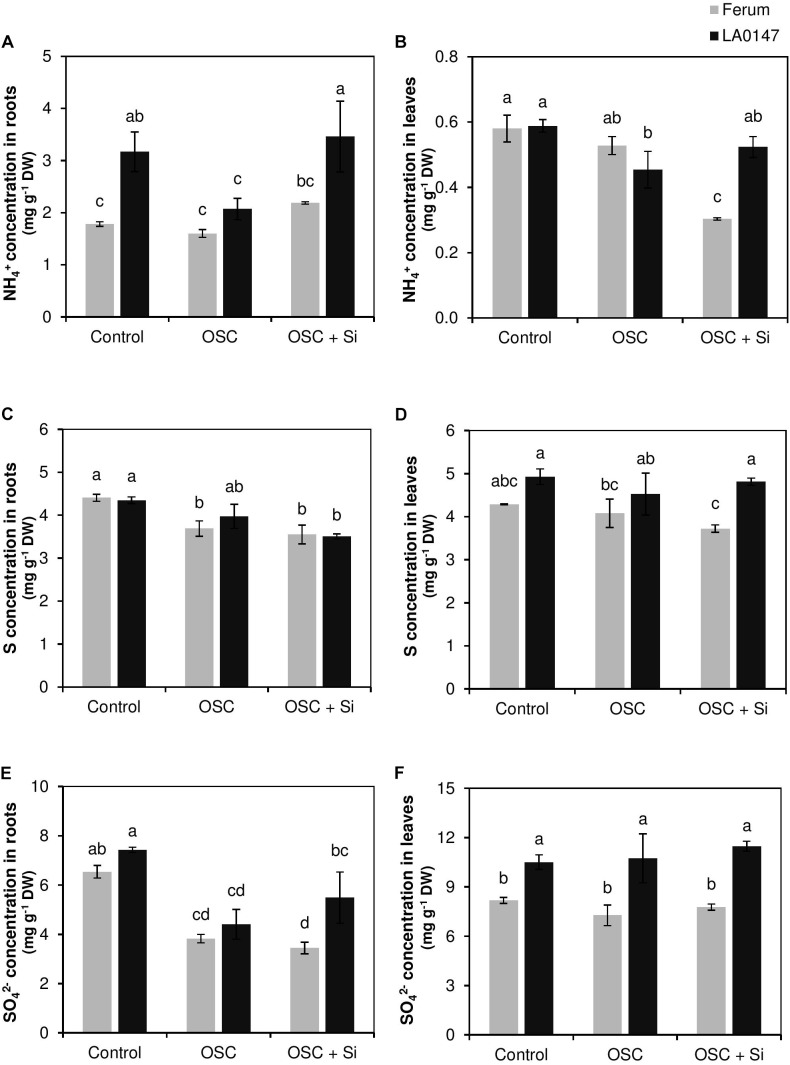
Influence of Si supply on ${\text{NH}}_{4}^{+}$, S, and ${\text{SO}}_{4}^{2-}$ concentrations in roots and leaves of two contrasting tomato genotypes under osmotic stress. **(A)** ${\text{NH}}_{4}^{+}$ concentration in roots, **(B)** ${\text{NH}}_{4}^{+}$ concentration in leaves, **(C)** S concentration in roots, **(D)** S concentration in leaves, **(E)** ${\text{SO}}_{4}^{2-}$ concentration in roots and **(F)** ${\text{SO}}_{4}^{2-}$ concentration in leaves. Plants were grown in hydroponic culture and osmotic stress was simulated by applying polyethylene glycol (PEG 6000). Si was provided at 0.75 mM for pre-cultured plants and at 1.5 mM for osmotic stressed plants. Roots and fully expanded leaves from 21-day old plants were harvested 7 days after imposition of osmotic stress. Bars indicate means ± SE. Different letters denote significant differences according to Fischer’s LSD test (*p* < 0.05; *n* = 4).

Elemental analysis revealed a distinct element profile under OSC among the two tested tomato lines, particularly with Si application. The root ${\text{NH}}_{4}^{+}$ concentrations was significantly higher in the tolerant line LA0147 in control condition in comparison to the sensitive line FERUM (**Figure 2A**). ${\text{NH}}_{4}^{+}$ level was similar in the shoots of both lines under control conditions (**Figure 2B**). We did not observe any particular changes in the level of ${\text{NH}}_{4}^{+}$ under OSC in either the tolerant line LA0147 or in the sensitive line FERUM (**Figures 2A,B**). Notably, ${\text{NH}}_{4}^{+}$ concentrations increased significantly in the tolerant line LA0147 in both roots and shoots under OSC by Si supply (**Figures 2A,B**). Relative to control plants, the root S concentrations were significantly decreased under OSC only in the sensitive line FERUM (**Figure 2C**). S concentration was not influenced by either osmotic stress or supplemental Si in shoots of both the lines (**Figure 2D**). Unlike ${\text{NH}}_{4}^{+}$, the level of root sulfate (${\text{SO}}_{4}^{2-}$) was similar in both lines under control conditions and ${\text{SO}}_{4}^{2-}$ remained significantly higher under control condition in the shoots of LA0147 (**Figures 2E,F**). There was no difference in root ${\text{SO}}_{4}^{2-}$ concentrations of either line under OSC, while shoot ${\text{SO}}_{4}^{2-}$ level was significantly increased in the tolerant line LA0147 compared to the sensitive line FERUM (**Figures 2E,F**).

### Metabolic Changes in Response to Osmotic Stress and Si Supply in Leaves of Both Tolerant and Sensitive Lines

The differential response of both the lines to osmotic stress was analyzed by comparing the metabolic changes in the roots and leaves of control, osmotic-stressed as well as osmotic-stressed plants exposed to Si supply. A total of 22 and 27 metabolites were determined in roots and leaves, respectively (**Supplementary Tables S2, S3**). Principal component analysis (PCA) revealed an obvious distinction in the metabolites in both the lines in all tested conditions (**Supplementary Figure S2**). Specifically in the leaves, a significant difference was observed between the response of two tomato genotypes, which led to a differential increase in the level of Arg, Met, serine (Ser), glycine (Gly), alanine (Ala), Pro, threonine (Thr), GABA, GSH and GSSG in tolerant line LA0147 and the sensitive line FERUM, under stress treatments with or without Si supply.

### Si Mediated Distinctive Changes in the Metabolites of Both Tolerant and Sensitive Tomato Lines Under OSC

**FIGURE 3:**
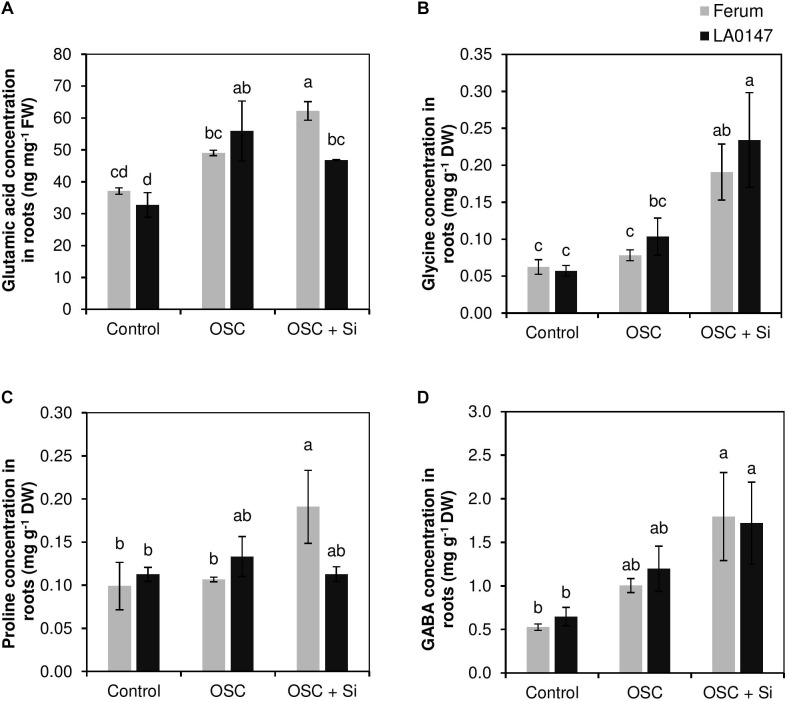
Influence of Si supply on selected metabolite concentrations in roots of two contrasting tomato genotypes under osmotic stress. **(A)** Glutamic acid concentration in roots, **(B)** Gly concentration in roots, **(C)** Pro concentration in roots, and **(D)** GABA concentration in roots. Plants were grown in hydroponic culture and osmotic stress was simulated by applying polyethylene glycol (PEG 6000). Si was provided at 0.75 mM for pre-cultured plants and at 1.5 mM for osmotic stressed plants. Roots and fully expanded leaves from 21-day old plants were harvested 7 days after imposition of osmotic stress. Bars indicate means ± SE. Different letters denote significant differences according to Fischer’s LSD test (*p* < 0.05; *n* = 4).

In the present study, Si resulted in a genotypic difference in metabolic profiles. In roots, only the level of glutamic acid as well as the level of amino acids Gly, Pro, and GABA did change (**Figure 3**). The level of glutamic acid was constant under control and increased when osmotic stress was applied. In osmotic stressed plants, Si increased the glutamic acid level in the sensitive line FERUM compared to the tolerant line LA0147 (**Figure 3A**). Si significantly increased the level of Gly under OSC in both lines in comparison to osmotic stressed plants which did not receive Si (**Figure 3B**). This increase was similar for Pro levels only in the sensitive line FERUM (**Figure 3C**). The GABA level was also significantly higher in OSC + Si plants when compared to control plants, however, in comparison to OSC, Si did not had any influence on the level of GABA in either of the tested genotypes (**Figure 3D**). The concentration of other metabolites in the roots did not show much variation (**Supplementary Table S2**).

**FIGURE 4:**
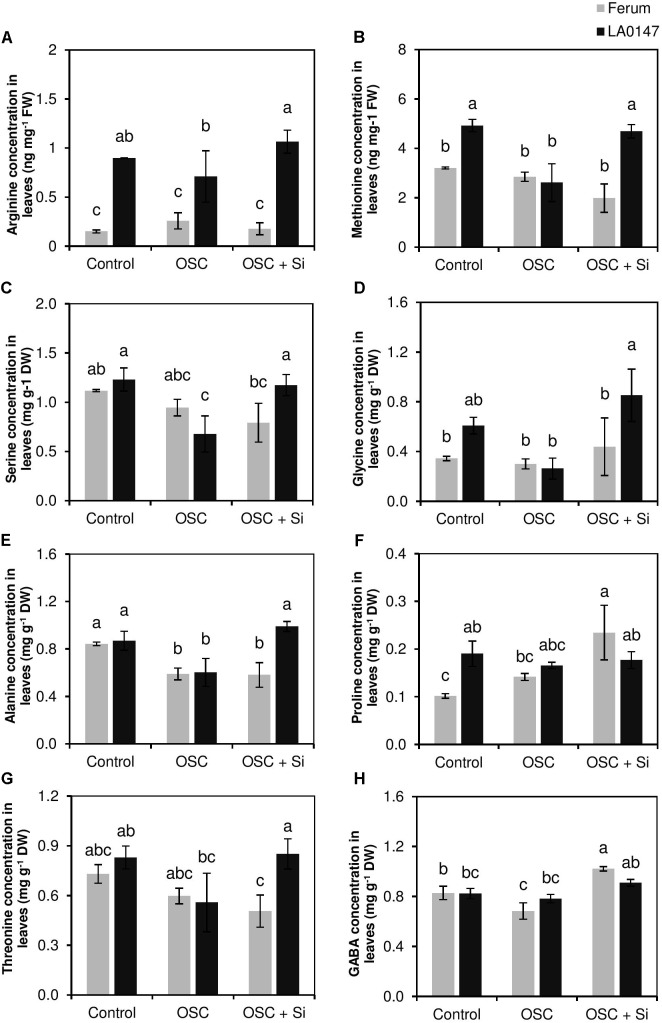
Influence of Si supply on selected amino acids concentration in leaves of two contrasting tomato genotypes under osmotic stress. **(A)** Arg concentration in leaves, **(B)** Met concentration in leaves, **(C)** Ser concentration in leaves, **(D)** Gly concentration in leaves, **(E)** Ala concentration in leaves, **(F)** Pro concentration in leaves, **(G)** Thr concentration in leaves, and **(H)** GABA concentration in leaves. Plants were grown in hydroponic culture and osmotic stress was simulated by applying polyethylene glycol (PEG 6000). Si was provided at 0.75 mM for pre-cultured plants and at 1.5 mM for osmotic stressed plants. Roots and fully expanded leaves from 21-day old plants were harvested 7 days after imposition of osmotic stress. Bars indicate means ± SE. Different letters denote significant differences according to Fischer’s LSD test (*p* < 0.05; *n* = 4). The units of Arg and Met are expressed in ng mg^-1^ FW.

In leaves, we observed a significant increase in the level of amino acids Arg, Met, and Gly in the tolerant line LA0147 compared to the sensitive line FERUM under osmotic stress when plant did receive Si (**Figure 4**). Compared to control plants, the level of amino acids Met, Ser, Gly were constant under OSC for the sensitive line FERUM, while osmotic stress reduced the levels of these amino acids in the tolerant line LA0147 (**Figures 4B–D**). Interestingly, under OSC, Si restored the level of amino acids Arg, Met, Gly, and Thr in the tolerant line LA0147 to the same level as control plants (**Figures 4A,B,D,G**). The increase in the level of amino acids under OSC by Si was also seen for Pro and GABA for both the lines, but compared to OSC, it was only significant in the sensitive line FERUM (**Figures 4F,H**). Among organic acids, concentration of malic acid was significantly increased by Si supply in the sensitive line under OSC compared to tolerant line LA0147 (**Supplementary Table S3**).

**FIGURE 5:**
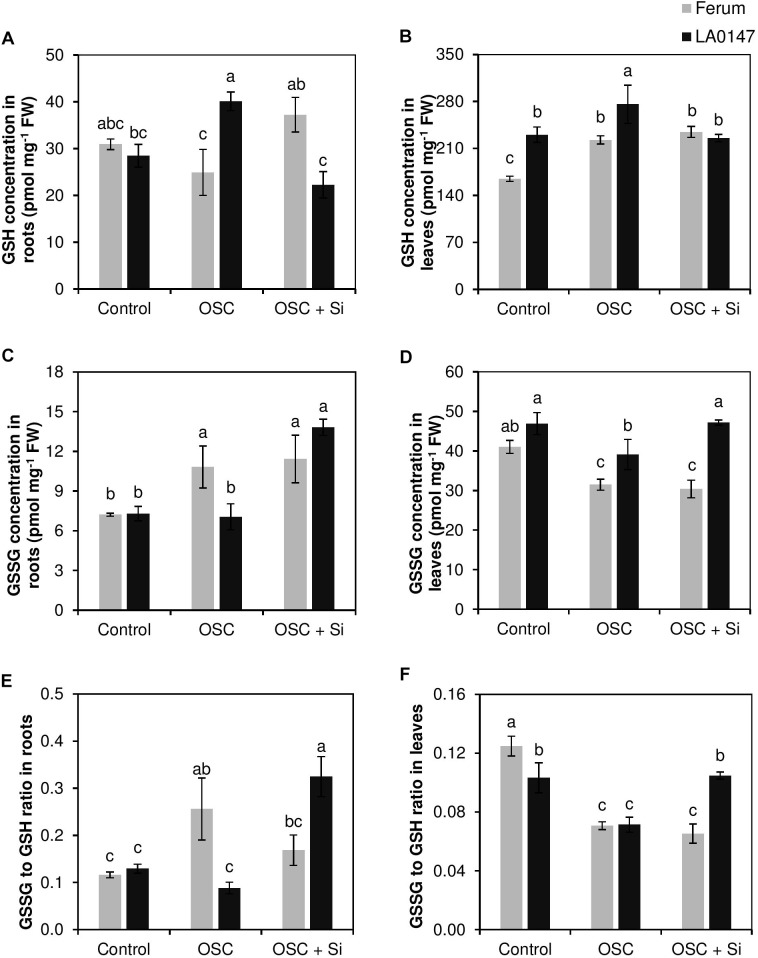
Influence of Si supply on glutathione concentrations in roots and leaves of two contrasting tomato genotypes under osmotic stress. **(A)** GSH concentration in roots, **(B)** GSH concentration in leaves, **(C)** GSSG concentration in roots, **(D)** GSSG concentration in leaves **(E)** GSSG to GSH ratio in roots and **(F)** GSSG to GSH ratio in leaves. Plants were grown in hydroponic culture and osmotic stress was simulated by applying polyethylene glycol (PEG 6000). Si was provided at 0.75 mM for pre-cultured plants and at 1.5 mM for osmotic stressed plants. Roots and fully expanded leaves from 21-day old plants were harvested 7 days after imposition of osmotic stress. Bars indicate means ± SE. Different letters denote significant differences according to Fischer’s LSD test (*p* < 0.05; *n* = 4).

We further measured the glutathione levels in both roots and leaves. In roots and under control conditions, the GSH, GSSG, and GSSG/GSH ratio was identical in both the lines (**Figures 5A,C,E**). While the level of GSH did not differ under OSC in the sensitive line FERUM compared to the control, osmotic stress significantly increased GSH level in the tolerant line LA0147 (**Figure 5A**). Si supply increased the GSH level in the sensitive line FERUM under OSC, whereas, its level decreased by Si in the tolerant line LA0147 (**Figure 5A**). Conversely, the GSSG level significantly increased under OSC in the sensitive line FERUM while it was constant for the tolerant line LA0147 (**Figure 5C**). The GSSG level was similar under OSC in both line when Si was added into the medium (**Figure 5C**) and was significantly higher in the tolerant line LA0147 compared to OSC (**Figure 5C**). The GSSG to GSH ratio which defines severity of oxidative stress was also calculated and was shown to be constant in either lines under control condition, however, this ratio significantly increased only in the sensitive line FERUM under OSC and in the tolerant line LA0147 under OSC + Si (**Figure 5E**).

In leaves, we observed a significantly higher concentration of GSH under both control and OSC in the tolerant line LA0147 compared to sensitive line FERUM (**Figure 5B**). Si did not influence the GSH levels under OSC in either of the investigated lines (**Figure 5B**). Similarly, the concentration of GSSG, the oxidized form of glutathione was higher in the tolerant line in almost all the tested conditions, and unlike GSH, Si resulted in significantly higher levels of GSSG under OSC in the tolerant line LA0147 (**Figure 5D**) which subsequently led to a significantly higher GSSG to GSH ratio in this line compared to the sensitive line FERUM (**Figure 5F**). These results suggests that the sensitive line underwent less oxidative stress compared to the tolerant line.

**FIGURE 6:**
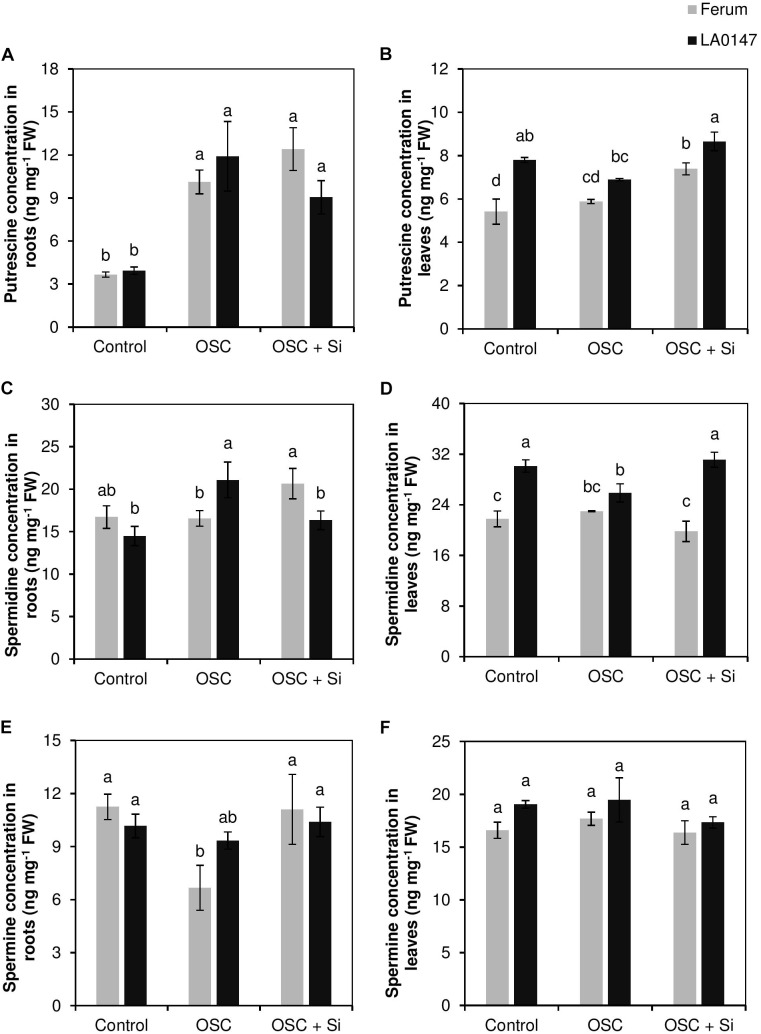
Influence of Si supply on polyamines concentrations in roots and leaves of two contrasting tomato genotypes under osmotic stress. **(A)** Put concentration in roots, **(B)** Put concentration in leaves, **(C)** spermidine concentration in roots, **(D)** spermidine concentration in leaves, **(E)** spermine concentration in roots, and **(F)** spermine concentration in leaves. Plants were grown in hydroponic culture and osmotic stress was simulated by applying polyethylene glycol (PEG 6000). Si was provided at 0.75 mM for pre-cultured plants and at 1.5 mM for osmotic stressed plants. Roots and fully expanded leaves from 21-day old plants were harvested 7 days after imposition of osmotic stress. Bars indicate means ± SE. Different letters denote significant differences according to Fischer’s LSD test (*p* < 0.05; *n* = 4).

It is well-known that the accumulation of polyamines results in drought stress tolerance. Therefore, the levels of polyamines putrescine (Put), spermidine, and spermine were also determined in both roots and leaves. In roots, Put showed significant increase in both the lines under OSC irrespective of Si supply, while spermidine increased significantly under OSC in the tolerant line only (**Figures 6A,C**). In the sensitive line FERUM, spermine levels significantly decreased under OSC as compared to control, however, Si supply under OSC further restored its level similar to the control (**Figure 6E**). In leaves and under control conditions, the levels of both Put and spermidine were significantly higher in LA0147 in comparison to FERUM (**Figures 6B,D**). Under OSC, the levels of these two polyamines were similar to the control plants in FERUM, however, significant reductions in the levels of spermidine were observed in LA0147 (**Figure 6D**). The level of Put and spermidine were both significantly higher under OSC + Si in LA0147 compared to FERUM (**Figures 6B,D**). Si also resulted in a significant increase in the level of Put in the sensitive line FERUM under OSC + Si compared to OSC (**Figure 6B**). There was no consistent change in the level of spermine in leaves of either lines (**Figure 6F**).

### Transcriptional Regulation of the Genes Involved in Arg, Met, and GABA Synthesis

**FIGURE 7:**
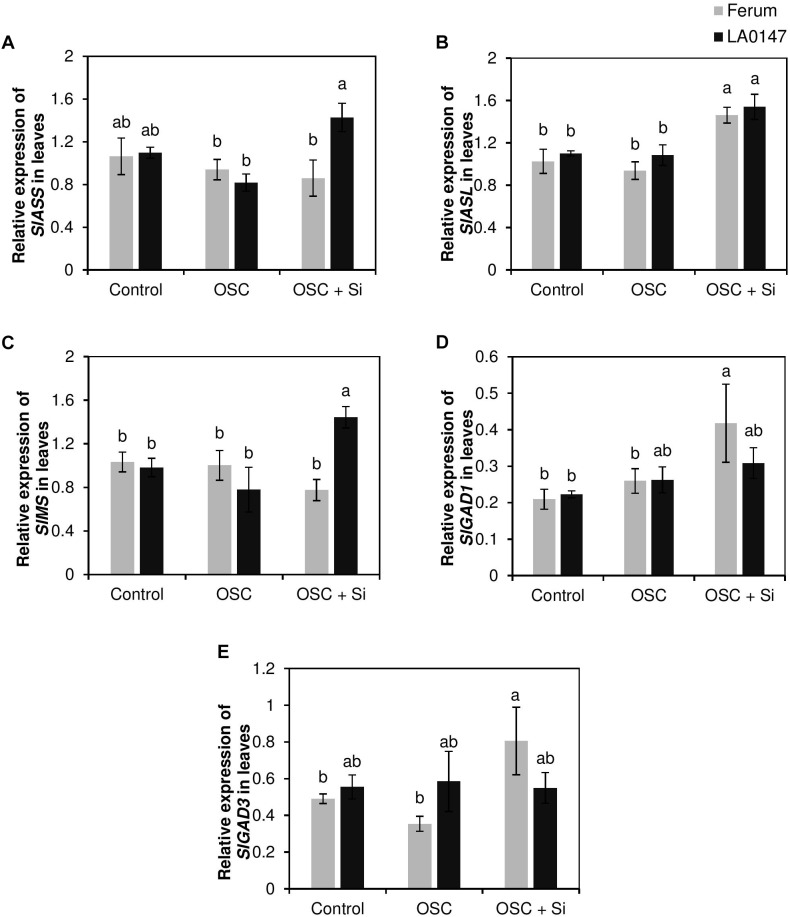
Influence of Si supply on expression levels of the genes involved in arginine, methionine, and GABA synthesis pathway in leaves of two contrasting tomato genotypes under osmotic stress. **(A)** relative expression of *SlASS*, **(B)** relative expression of *SlASL*, **(C)** relative expression of *SlMS*, **(D)** relative expression of *SlGAD1*, and **(E)** relative expression of *SlGAD3*. Plants were grown in hydroponic culture and osmotic stress was simulated by applying polyethylene glycol (PEG 6000). Si was provided at 0.75 mM for pre-cultured plants and at 1.5 mM for osmotic stressed plants. Roots and fully expanded leaves from 21-day old plants were harvested 7 days after imposition of osmotic stress. Bars indicate means ± SE. Different letters denote significant differences according to Fischer’s LSD test (*p* < 0.05; *n* = 4).

The higher Arg and Met levels in the tolerant line LA0147 further encouraged us to investigate whether Si exerted any impact on the transcription of the genes involved in the synthesis of the two amino acids Arg and Met. Monitoring the expression level of the genes encoding argininosuccinate synthase (*SlASS*) and argininosuccinate lyase (*SlASL*) which are involved in the Arg synthesis pathway, showed a significant induction of *SlASS* only in LA0147 by Si under OSC in comparison to FERUM (**Figure 7A**). The expression level of *SlASL* was however induced in both lines when treated with Si and subjected to OSC (**Figure 7B**). The expression level of methionine synthase (*SlMS*) which is involved in Met synthesis pathway, displayed a significant induction in the tolerant line LA0147 when plants were exposed to OSC and supplemented with Si (OSC + Si) (**Figure 7C**).

In addition, transcript levels of the genes involved in GABA, Pro synthesis and GSH recycling were analyzed. We did not observe any consistent change in the expression pattern of the genes involved in the Pro and GSH pathways (**Supplementary Figure S3**). Interestingly, Si significantly increased the expression levels of glutamate decarboxylase genes (*SlGAD1* and *SlGAD3*) which converts glutamate into GABA (Kamei et al., 2011) under OSC only in the drought-sensitive line FERUM (**Figures 7D,E**). These results show that Si transcriptionally regulates the genes involved in Arg and Met synthesis in the tolerant line LA0147 and GABA synthesis in the sensitive line FERUM when plants were subjected to osmotic stress.

## Discussion

There is an increasing research interest on Si nutrition (Cooke and Leishman, 2016; Neu et al., 2017). Particularly during the last decade, numerous studies have demonstrated the beneficial effect of Si in alleviating adverse responses to abiotic stresses and mineral nutrition deficiencies (Cooke and Leishman, 2016; Neu et al., 2017). Ma et al. (2016) showed a positive effect of Si on drought-stressed winter wheat plants by regulating the antioxidant systems (Ma et al., 2016). Si was shown to delay drought-induced leaf senescence in *Sorghum* by increasing polyamine levels and decreasing ACC concentration (Yin et al., 2014). In another study, Si was shown to mitigate stress in rice plants imposed to drought by contributing to plant water uptake, improving photosynthesis and an improved absorption of minerals (Chen et al., 2011). None of the previous studies made a direct comparison of drought tolerant and drought sensitive crop cultivars in response to Si nutrition. In the present study, investigating the role of Si in two tomato lines contrasting in their response to drought stress we found a differential response of each genotype in response to PEG-induced osmotic stress. While drought tolerant line LA0147 showed better growth responses under OSC and further maintained it upon Si supply, the sensitive line FERUM displayed an improvement in the growth parameters under OSC only when Si was supplied to the plants (**Figures 1A–E**). This interesting observation encouraged us to further investigate the mechanism (s) by which these two lines responds to osmotic stress particularly when they received supplemental Si.

**FIGURE 8:**
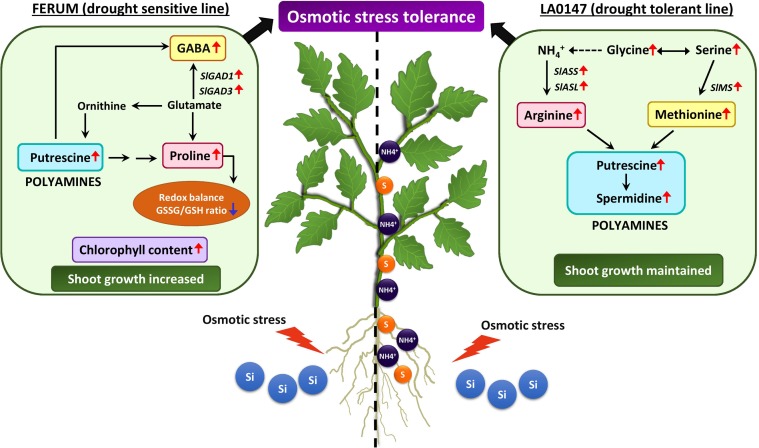
Schematic model demonstrating the distinct regulatory role of Si in mediating differential stress tolerance responses in two contrasting tomato lines exposed to osmotic stress. Under osmotic stress, the two tomato genotypes LA0147 (drought tolerant) and FERUM (drought sensitive) showed differential stress tolerance responses facilitated by supplemental Si. In LA0147, under osmotic stress, application of Si mediated increased uptake and translocation of ${\text{NH}}_{4}^{+}$ and S, which led to consequent alterations in the accumulation of different amino acids like arginine, methionine, serine, and glycine and regulation of genes involved in Arg and Met synthesis. An upsurge of these amino acids further resulted in augmentation of polyamines putrescine and spermidine which maintained shoot growth and other stress tolerance responses. On the other hand, Si supply provided to the osmotic stressed plants of sensitive line FERUM showed a completely different pattern in accumulation of specific amino acids proline and GABA, which are known to provide better stress tolerance in plants by balancing the redox homeostasis with evident decrease in GSSG/GSH ratio. The higher accumulation of GABA was caused by Si induced expression of *SlGAD1* and *SlGAD3*, which are involved in GABA synthesis. A simultaneous increase in the levels specifically of the free polyamine Put was also observed in FERUM which subsequently promoted an improved osmotic stress tolerance as suggested by increased shoot growth and higher chlorophyll content (Red arrows = increase/upregulation, blue arrows = decrease/downregulation).

An early consequence of the low water availability under drought is a decrease in total nutrient uptake and translocation to shoots (Hosseini et al., 2016). Si was shown to improve the uptake and translocation of different individual minerals in crops (Pavlovic et al., 2013). For instance, in *Sorghum* seedlings grown in potassium deficient condition, Si enhanced the concentration of potassium in both roots and shoots (Miao et al., 2010). Also in cucumber, Si increased leaf remobilization of iron under iron deficiency (Pavlovic et al., 2016). In the current study, Si resulted in a higher uptake and translocation of ${\text{NH}}_{4}^{+}$ and S under OSC only in the tolerant line LA0147 compared to the sensitive line FERUM (**Figure 2**) indicating that Si contributed efficiently to a better ${\text{NH}}_{4}^{+}$ and S uptake in the tolerant line LA0147 under OSC. Several lines of evidence suggests that translocation of ${\text{NH}}_{4}^{+}$ from roots to shoots is mediated by specific transport systems which are capable of loading ${\text{NH}}_{4}^{+}$ into the xylem (von Wiren et al., 2000; Loqué and von Wirén, 2004). However, the molecular aspects of ${\text{NH}}_{4}^{+}$ transport in the shoots or leaf cells have been investigated to a limited extent (Schjoerring et al., 2002). In a more recent investigation by Haddad et al. (2018), the authors clearly demonstrated that Si treatments augmented the translocation of nitrate in *Brassica napus* which was associated with higher expression of nitrate transporter genes. Therefore, in the present study it could be assumed that Si treatments might have caused the differential response of tolerant and sensitive lines to ${\text{NH}}_{4}^{+}$ translocation in the roots and shoots. However, further investigations are needed to understand the exact role Si plays in translocation of ${\text{NH}}_{4}^{+}$ from roots to shoots using different sources of nitrogen.

To further investigate whether the Si-mediated increase in the levels of S and ${\text{NH}}_{4}^{+}$ are involved in the differential response of these two lines under OSC, metabolite profiling was performed which showed a distinct change among the two lines under OSC especially when these lines were treated with Si (**Figures 2A,B**). Interestingly, our results revealed that Si positively contributed in altering the levels of selected amino acids and polyamines which are known to be involved in drought stress responses in plants and improves tolerance. In the leaves of the tolerant line LA0147, the levels of Arg, Met, Ser, Ala, and Gly increased under OSC when Si was supplied (**Figure 4**). Both Arg and Met are involved in the synthesis pathways for polyamines which play an important role under drought stress (Hesse et al., 2004; Slocum, 2005). Ser also participates in the synthesis of amino acids cysteine (Cys) and Met which are involved in the S metabolism pathway (Wirtz and Droux, 2005). Serine is involved in the last step of Cys biosynthesis where the synthesis of the carbon/nitrogen-backbone of cysteine is catalyzed by serine acetyltransferase (SAT), which transfers an acetyl-moiety from acetyl-Coenzyme A (acetyl-CoA) to serine, leading to *O*-acetylserine (OAS) formation. OAS is then converted into Cys in the presence of sulfide (Wirtz and Droux, 2005). Cys is further converted to Met via cystathionine γ-synthase (CGS) and cystathionine β-lyase (CBL) which are involved in methionine biosynthesis in the chloroplasts (Ravanel et al., 2004). The S-based metabolites and their role in drought stress tolerance has been reported before in several studies (Chen et al., 2012; Li et al., 2013). Interestingly, in the present study, Si treatment resulted in a significantly higher production of the amino acid Gly in both roots and leaves of the tolerant line LA0147 under OSC (**Figures 3B**, **4D**). Glycine has been reported to play a crucial role in increasing tolerance of plants to stress conditions (Tripathi et al., 2013). It acts as a direct precursor of ${\text{NH}}_{4}^{+}$ and is also involved in increasing the level of Ser (Schiller et al., 1998; Waditee et al., 2005). It is worth noting that in the present study, the higher production of Met by Si was associated with a significantly higher level of amino acids Gly and Ser (**Figures 4C,D**). We also observed a significantly higher production of polyamines Put and spermidine in the leaves of the tolerant line LA0147 under OSC when Si was added to the medium (**Figures 6B,D**), and this could be due to a higher production of both amino acids Arg and Met (**Figures 4A,B**) (Liu et al., 2015). In agreement with these results Detmann et al. (2012b) observed a strong correlation between Si concentration and amino acids Arg and Met in the leaves of rice plants treated with Si. Additionally, in line with our finding, Yin et al. (2013) have shown that Si enhanced drought tolerance in *Sorghum bicolor* plants by interfering the balance between polyamines and ethylene levels. These authors reported a higher polyamines levels in favor of low ethylene production in S*orghum bicolor* plants which consequently delayed leaf senescence and increased drought tolerance (Yin et al., 2014). Monitoring the expression levels of *SlASS*, *SlASL* (Arg synthesis) and *SlMS* (Met synthesis) also confirmed that Si regulated the Arg and Met synthesis pathways at the transcriptional level (**Figure 7**). Altogether, these results suggest that Si coordinates stress tolerance in the drought tolerant line LA0147 by increasing uptake of ${\text{NH}}_{4}^{+}$ and S which led to significantly higher synthesis of the amino acids Arg and Met by regulating the genes involved in their synthesis pathways. This further enhanced the level of polyamines Put and spermidine which finally maintained tolerance to osmotic stress in this line.

On the other hand, in both roots and leaves Si resulted in significantly higher production of amino acids Pro and GABA under OSC + Si in the sensitive line FERUM (**Figures 3C,D**, **4F,H**). The higher production of GABA and Pro are a common physiological response in higher plants exposed to drought stress. These two amino acids are synthesized from glutamic acid which is the main pathway under drought conditions for the production of Pro and GABA (Liu et al., 2011). It is well documented that reactive oxygen species (ROS) levels increase under drought stress resulting in severe damage of DNA, RNA, protein, and membrane oxidation and finally damage of cell structure which then turns to oxidative stress (Noctor et al., 2014). ROS is detoxified by the antioxidant systems that include superoxide dismutases (SODs), glutathione peroxidases (GPXs), peroxiredoxins (PRXs), and other antioxidants such as glutathione (GSH) (Mittler, 2017). Likewise, accumulated osmolytes under drought stress are known to contribute to the scavenging of ROS in plants. Many studies have reported Pro as a ROS scavenger (Pospíšil, 2012). In addition, under stress conditions, a GSH-based defense system is activated to maintain lower levels of cellular redox by reducing the level of varied ROS (Anjum et al., 2014). Interestingly, in this study, Si resulted in significantly lower GSSG/GSH ratio in both roots and leaves under OSC in the sensitive line FERUM compared to tolerant line LA0147 (**Figures 5E,F**), indicating the higher capacity of sensitive line FERUM to balance cellular redox homeostasis to mitigate oxidative stress under OSC when Si was supplied. Under drought conditions, the higher GSSG to GSH ratio has been often reported (Foyer and Noctor, 2011; Chan et al., 2013). The higher conversion of GSH toward the oxidized form GSSG result in severe oxidative stress in maize plants exposed to drought stress (Ahmad et al., 2016). In line with these results, several studies have shown that application of Si mitigated oxidative stress by modulating the antioxidant enzymes in soybean (Shen et al., 2010), wheat (Gong et al., 2008) and chickpea (Gunes et al., 2007). A higher production of Pro has also been reported in drought sensitive tomato (Schafleitner et al., 2007). Notably, the concentration of free Put was also higher in the sensitive line FERUM under OSC when treated with Si (**Figure 6B**) indicating the capacity of sensitive line FERUM to improve osmotic stress tolerance by accumulation of free polyamines following Si application. Accumulation of polyamines mediated by supplemental Si in response to drought stress has been extensively reported in several studies (Chen et al., 2013; Yin et al., 2013; Sui et al., 2015).

It is worth noting that in the leaves, GABA accumulated significantly under OSC in the sensitive line FERUM treated with Si (**Figure 4H**). The function of GABA in ROS scavenging has been shown in *Nicotiana tabacum* (Liu et al., 2011) and black cumin (Rezaei-Chiyaneh et al., 2018) exposed to water stress. As four-carbon, non-protein amino acid, GABA is found in both prokaryotes and eukaryotes. GABA is synthesized from glutamic acid and is synthesized under stress conditions in plants (Liu et al., 2011). In a study in white clover, application of GABA to plants exposed to PEG-induced drought stress was associated with an increase in the level of the free polyamines Put and spermidine and a higher production of proline which led to improved drought tolerance (Rezaei-Chiyaneh et al., 2018). It has also been shown that application of GABA to *Nicotiana tabacum* leaves exposed to drought stress reduced the level of ROS even more effectively than proline (Liu et al., 2011). Therefore, an increase in GABA level in the sensitive line FERUM was associated with a higher production of free polyamine Put which contributes to improved stress tolerance in this line. Furthermore, the gene expression analysis also showed that Si transcriptionally regulates the expression levels of both *SlGAD1* and *SlGAD3* under OSC in the sensitive line FERUM (**Figures 7D,E**). Glutamate decarboxylase (GAD) converts glutamate into GABA and is also identified as an aging gene which was shown to be required for normal oxidative stress in the budding yeast *Saccharomyces cerevisiae* (Coleman et al., 2001; Kamei et al., 2011). In addition, in soil-grown Arabidopsis plants subjected to salinity stress, the accumulation of GABA was associated with upregulation of the GAD genes *GAD1*, *GAD2*, and *GAD4* (Zarei et al., 2017). Therefore, it can be concluded that the higher GABA level under OSC in the sensitive line FERUM is caused by Si-induced expression of GABA related genes. Together, these results suggest a mechanism by which Si co-ordinately enhances osmotic stress tolerance in the sensitive line FERUM by leading to a significantly higher synthesis of the amino acids Pro and GABA. This then leads to a reduction in GSSG levels in order to balance redox homeostasis and increases the level of free polyamine Put to cope better with osmotic stress as supported by higher biomass and higher chlorophyll index (**Figures 1B–E**).

## Conclusion

We believe that the current study is the first to report a direct comparison of drought tolerant and drought sensitive crop cultivars in response to Si nutrition. We demonstrated two distinct regulatory roles for Si in drought sensitive and drought tolerant tomato lines in order to cope with osmotic stress. In the tolerant line LA0147, Si results in a higher uptake and translocation of ${\text{NH}}_{4}^{+}$ and S under OSC. An enhanced uptake of these two elements contributes to a significantly higher production of amino acids Arg and Met by Si under OSC which further results in a higher production of polyamines Put and spermidine thus maintaining the growth and increasing osmotic stress tolerance. In the sensitive line FERUM, Si primarily influences the accumulation of amino acids Pro and GABA under OSC, subsequently leading to a lower GSSG/GSH ratio and higher production of polyamine Put under OSC (**Figure 8**). Therefore, it appears that Si positively contributes to an improved osmotic stress tolerance in the sensitive line FERUM by restoring growth and chlorophyll levels. Si also transcriptionally regulates the genes involved in Arg, Met, and GABA synthesis under OSC in drought tolerant and drought sensitive lines, respectively. We conclude that beneficial effect of Si were clearly visible in the sensitive line FERUM compared to tolerant line LA0147. Si predominantly regulates osmotic stress in both lines by distinct regulation of relevant amino acids which are involved in stress responses in the plant. The involvement of polyamine metabolism was a shared response of both lines to osmotic stress after treating with Si.

## Author Contributions

NA conducted the experiments and analyzed data. AS performed the metabolite analysis. NA performed gene expression analysis. SH designed the experiments and evaluated the data. SH, J-CY, and NA wrote the article.

## Conflict of Interest Statement

The authors declare that the research was conducted in the absence of any commercial or financial relationships that could be construed as a potential conflict of interest.

## References

[B1] Abdallah M. Dubousset L. Meuriot F. Etienne P. Avice J. C. Ourry A. (2010). Effect of mineral sulphur availability on nitrogen and sulphur uptake and remobilization during the vegetative growth of *Brassica napus* L. *J. Exp. Bot.* 61 2635–2646. 10.1093/jxb/erq096 20403880 PMC2882259

[B2] Ahmad N. Malagoli M. Wirtz M. Hell R. (2016). Drought stress in maize causes differential acclimation responses of glutathione and sulfur metabolism in leaves and roots. *BMC Plant Biol.* 16:247. 10.1186/s12870-016-0940-z 27829370 PMC5103438

[B3] Amin M. Ahmad R. Ali A. Hussain I. Mahmood R. Aslam M. (2016). Influence of silicon fertilization on maize performance under limited water supply. *Silicon* 10 177–183. 10.1007/s12633-015-9372-x

[B4] Amtmann A. Armengaud P. (2009). Effects of N, P, K and S on metabolism: new knowledge gained from multi-level analysis. *Curr. Opin. Plant Biol.* 12 275–283. 10.1016/j.pbi.2009.04.014 19493694

[B5] Anjum N. A. Aref I. M. Duarte A. C. Pereira E. Ahmad I. Iqbal M. (2014). Glutathione and proline can coordinately make plants withstand the joint attack of metal(loid) and salinity stresses. *Front. Plant Sci.* 5:662. 10.3389/fpls.2014.00662 25484889 PMC4240066

[B6] Armengaud P. Sulpice R. Miller A. J. Stitt M. Amtmann A. Gibon Y. (2009). Multilevel analysis of primary metabolism provides new insights into the role of potassium nutrition for glycolysis and nitrogen assimilation in *Arabidopsis* roots. *Plant Physiol.* 150 772–785. 10.1104/pp.108.133629 19346439 PMC2689955

[B7] Cao B. L. Wang L. Gao S. Xia J. Xu K. (2017). Silicon-mediated changes in radial hydraulic conductivity and cell wall stability are involved in silicon-induced drought resistance in tomato. *Protoplasma* 254 2295–2304. 10.1007/s00709-017-1115-y 28536765

[B8] Chan K. X. Wirtz M. Phua S. Y. Estavillo G. M. Pogson B. J. (2013). Balancing metabolites in drought: the sulfur assimilation conundrum. *Trends Plant Sci.* 18 18–29. 10.1016/j.tplants.2012.07.005 23040678

[B9] Chen J.-H. Jiang H.-W. Hsieh E.-J. Chen H.-Y. Chien C.-T. Hsieh H.-L. (2012). Drought and salt stress tolerance of an *Arabidopsis* glutathione S-transferase U17 knockout mutant are attributed to the combined effect of glutathione and abscisic acid. *Plant Physiol.* 158 340–351. 10.1104/pp.111.181875 22095046 PMC3252094

[B10] Chen T. Xu Y. Wang J. Wang Z. Yang J. Zhang J. (2013). Polyamines and ethylene interact in rice grains in response to soil drying during grain filling. *J. Exp. Bot.* 64 2523–2538. 10.1093/jxb/ert115 23606413

[B11] Chen W. Yao X. Cai K. Chen J. (2011). Silicon alleviates drought stress of rice plants by improving plant water status, photosynthesis and mineral nutrient absorption. *Biol. Trace Elem. Res.* 142 67–76. 10.1007/s12011-010-8742-x 20532668

[B12] Coleman S. T. Fang T. K. Rovinsky S. A. Turano F. J. Moye-Rowley W. S. (2001). Expression of a glutamate decarboxylase homologue is required for normal oxidative stress tolerance in *Saccharomyces cerevisiae*. *J. Biol. Chem.* 276 244–250. 10.1074/jbc.M007103200 11031268

[B13] Cooke J. Leishman M. R. (2016). Consistent alleviation of abiotic stress with silicon addition: a meta-analysis. *Funct. Ecol.* 30 1340–1357. 10.1111/1365-2435.12713

[B14] Detmann K. C. Araújo W. L. Martins S. C. Sanglard L. M. Reis J. V. Detmann E. (2012a). Silicon nutrition increases grain yield, which, in turn, exerts a feed-forward stimulation of photosynthetic rates via enhanced mesophyll conductance and alters primary metabolism in rice. *New Phytol.* 196 752–762. 10.1111/j.1469-8137.2012.04299.x 22994889

[B15] Detmann K. C. Araújo W. L. Martins S. C. V. Fernie A. R. Damatta F. M. (2012b). Metabolic alterations triggered by silicon nutrition: is there a signaling role for silicon? *Plant Signal. Behav.* 8:e22523. 10.4161/psb.22523 23104113 PMC3745559

[B16] Expósito-Rodríguez M. Borges A. A. Borges-Pérez A. Pérez J. A. (2008). Selection of internal control genes for quantitative real-time RT-PCR studies during tomato development process. *BMC Plant Biol.* 8:131. 10.1186/1471-2229-8-131 19102748 PMC2629474

[B17] Foyer C. H. Noctor G. (2011). Ascorbate and glutathione: the heart of the redox hub. *Plant Physiol.* 155 2–18. 10.1104/pp.110.167569 21205630 PMC3075780

[B18] Ghosson H. Schwarzenberg A. Jamois F. Yvin J. C. (2018). Simultaneous untargeted and targeted metabolomics profiling of underivatized primary metabolites in sulfur- deficient barley by ultra-high performance liquid chromatography-quadrupole/time- of-flight mass spectrometry. *Plant Methods* 14:62. 10.1186/s13007-018-0329-0 30061918 PMC6056915

[B19] Gimeno V. Díaz-López L. Simón-Grao S. Martínez V. Martínez-Nicolás J. J. García-Sánchez F. (2014). Foliar potassium nitrate application improves the tolerance of *Citrus macrophylla* L. seedlings to drought conditions. *Plant Physiol. Biochem.* 83 308–315. 10.1016/j.plaphy.2014.08.008 25218731

[B20] Gong H. J. Chen K. M. Zhao Z. G. Chen G. C. Zhou W. J. (2008). Effects of silicon on defense of wheat against oxidative stress under drought at different developmental stages. *Biol. Plant.* 52 592–596. 10.1007/s10535-008-0118-0

[B21] Gunes A. Pilbeam D. J. Inal A. Bagci E. G. Coban S. (2007). Influence of silicon on antioxidant mechanisms and lipid peroxidation in chickpea (*Cicer arietinum* L.) cultivars under drought stress. *J. Plant Interact.* 2 105–113. 10.1080/17429140701529399

[B22] Haddad C. Arkoun M. Jamois F. Schwarzenberg A. Yvin J. C. Etienne P. (2018). Silicon promotes growth of *Brassica napus* L. and delays leaf senescence induced by nitrogen starvation. *Front. Plant Sci.* 9:516. 10.3389/fpls.2018.00516 29740460 PMC5925743

[B23] Hesse H. Kreft O. Maimann S. Zeh M. Hoefgen R. (2004). Current understanding of the regulation of methionine biosynthesis in plants. *J. Exp. Bot.* 55 1799–1808. 10.1093/jxb/erh139 15234989

[B24] Hosseini S. A. Hajirezaei M. R. Seiler C. Sreenivasulu N. von Wirén N. (2016). A potential role of flag leaf potassium in conferring tolerance to drought-induced leaf senescence in barley. *Front. Plant Sci.* 7:206. 10.3389/fpls.2016.00206 26955376 PMC4768371

[B25] Hosseini S. A. Maillard A. Hajirezaei M. R. Ali N. Schwarzenberg A. Jamois F. (2017). Induction of barley silicon transporter hvlsi1 and hvlsi2, increased silicon concentration in the shoot and regulated starch and aba homeostasis under osmotic stress and concomitant potassium deficiency. *Front. Plant Sci.* 8:1359. 10.3389/fpls.2017.01359 28824688 PMC5541011

[B26] Ijaz R. Ejaz J. Gao S. Liu T. Imtiaz M. Ye Z. (2017). Overexpression of annexin gene AnnSp2, enhances drought and salt tolerance through modulation of ABA synthesis and scavenging ROS in tomato. *Sci. Rep.* 7:12087. 10.1038/s41598-017-11168-2 28935951 PMC5608957

[B27] Kamei Y. Tamura T. Yoshida R. Ohta S. Fukusaki E. Mukai Y. (2011). GABA metabolism pathway genes. *Biochem. Biophys. Res. Commun.* 407 185–190. 10.1016/j.bbrc.2011.02.136 21371425

[B28] Kang Y. Han Y. Torres-Jerez I. Wang M. Tang Y. Monteros M. (2011). System responses to long-term drought and re-watering of two contrasting alfalfa varieties. *Plant J.* 68 871–889. 10.1111/j.1365-313X.2011.04738.x 21838776

[B29] Li Y. Zhang J. Zhang J. Hao L. Hua J. Duan L. (2013). Expression of an *Arabidopsis* molybdenum cofactor sulphurase gene in soybean enhances drought tolerance and increases yield under field conditions. *Plant Biotechnol. J.* 11 747–758. 10.1111/pbi.12066 23581509

[B30] Liu C. Zhao L. Yu G. (2011). The dominant glutamic acid metabolic flux to produce y-amino butyric acid over proline in *Nicotiana tabacum* leaves under water stress relates to its significant role in antioxidant activity. *J. Integr. Plant Biol.* 53 608–618. 10.1111/j.1744-7909.2011.01049.x 21564543

[B31] Liu J.-H. Wang W. Wu H. Gong X. Moriguchi T. (2015). Polyamines function in stress tolerance: from synthesis to regulation. *Front. Plant Sci.* 6:827. 10.3389/fpls.2015.00827 26528300 PMC4602114

[B32] Loqué D. von Wirén N. (2004). Regulatory levels for the transport of ammonium in plant roots. *J. Exp. Bot.* 55 1293–1305. 10.1093/jxb/erh147 15133056

[B33] Ma C. C. Li Q. F. Gao Y. B. Xin T. R. (2004). Effects of silicon application on drought resistance of cucumber plants. *Soil Sci. Plant Nutr.* 50 623–632. 10.1080/00380768.2004.10408520

[B34] Ma D. Sun D. Wang C. Qin H. Ding H. Li Y. (2016). Silicon application alleviates drought stress in wheat through transcriptional regulation of multiple antioxidant defense pathways. *J. Plant Growth Regul.* 35 1–10. 10.1007/s00344-015-9500-2

[B35] Miao B. H. Han X. G. Zhang W. H. (2010). The ameliorative effect of silicon on soybean seedlings grown in potassium-deficient medium. *Ann. Bot.* 105 967–973. 10.1093/aob/mcq063 20338952 PMC2876006

[B36] Mittler R. (2017). ROS are good. *Trends Plant Sci.* 22 11–19. 10.1016/j.tplants.2016.08.002 27666517

[B37] Monti A. Brugnoli E. Scartazza A. Amaducci M. T. (2006). The effect of transient and continuous drought on yield, photosynthesis and carbon isotope discrimination in sugar beet (*Beta vulgaris* L.). *J. Exp. Bot.* 57 1253–1262. 10.1093/jxb/erj091 16467409

[B38] Nagy Z. Nemeth E. Guoth A. Bona L. Wodala B. Pecsvaradi A. (2013). Metabolic indicators of drought stress tolerance in wheat: glutamine synthetase isoenzymes and rubisco. *Plant Physiol. Biochem.* 67 48–54. 10.1016/j.plaphy.2013.03.001 23542183

[B39] Neu S. Schaller J. Dudel E. G. Ma J. F. Yamaji N. Struyf E. (2017). Silicon availability modifies nutrient use efficiency and content, C:N:P stoichiometry, and productivity of winter wheat (*Triticum aestivum* L.). *Sci. Rep.* 7:40829. 10.1038/srep40829 28094308 PMC5240101

[B40] Noctor G. Mhamdi A. Foyer C. H. (2014). The roles of reactive oxygen metabolism in drought: not so cut and dried. *Plant Physiol.* 164 1636–1648. 10.1104/pp.113.233478 24715539 PMC3982730

[B41] Pavlovic J. Samardzic J. Kostic L. Laursen K. H. Natic M. Timotijevic G. (2016). Silicon enhances leaf remobilization of iron in cucumber under limited iron conditions. *Ann. Bot.* 118 271–280. 10.1093/aob/mcw105 27371693 PMC4970368

[B42] Pavlovic J. Samardzic J. Maksimovič V. Timotijevic G. Stevic N. Laursen K. H. (2013). Silicon alleviates iron deficiency in cucumber by promoting mobilization of iron in the root apoplast. *New Phytol.* 198 1096–1107. 10.1111/nph.12213 23496257

[B43] Pei Z. F. Ming D. F. Liu D. Wan G. L. Geng X. X. Gong H. J. (2010). Silicon improves the tolerance to water-deficit stress induced by polyethylene glycol in wheat (*Triticum aestivum* L.) Seedlings. *J. Plant Growth Regul.* 29 106–115. 10.1007/s00344-009-9120-9

[B44] Pospíšil P. (2012). Molecular mechanisms of production and scavenging of reactive oxygen species by photosystem II. *Biochim. Biophys. Acta* 1817 218–231. 10.1016/J.BBABIO.2011.05.017 21641332

[B45] Ramegowda V. Basu S. Krishnan A. Pereira A. (2014). Rice growth under drought kinase is required for drought tolerance and grain yield under normal and drought stress conditions. *Plant Physiol.* 166 1634–1645. 10.1104/pp.114.248203 25209982 PMC4226359

[B46] Ravanel S. Block M. A. Rippert P. Jabrin S. Curien G. Rébeillé F. (2004). Methionine metabolism in plants: chloroplasts are autonomous for de novo methionine synthesis and can import S-adenosylmethionine from the cytosol. *J. Biol. Chem.* 279 22548–22557. 10.1074/jbc.M313250200 15024005

[B47] Rezaei-Chiyaneh E. Seyyedi S. M. Ebrahimian E. Moghaddam S. S. Damalas C. A. (2018). Exogenous application of gamma-aminobutyric acid (GABA) alleviates the effect of water deficit stress in black cumin (*Nigella sativa* L.). *Ind. Crops Prod.* 112 741–748. 10.1016/j.indcrop.2017.12.067

[B48] Schafleitner R. Gaudin A. Gutierrez Rosales R. O. Alvarado Aliaga C. A. Bonierbale M. (2007). Proline accumulation and real time PCR expression analysis of genes encoding enzymes of proline metabolism in relation to drought tolerance in Andean potato. *Acta Physiol. Plant.* 29 19–26. 10.1007/s11738-006-0003-4

[B49] Schiller P. Heilmeier H. Hartung W. (1998). Uptake of amino acids by the aquatic resurrection plant Chamaegigas intrepidus and its implication for N nutrition. *Oecologia* 117 63–69. 10.1007/s004420050632 28308507

[B50] Schjoerring J. K. Husted S. Mäck G. Mattsson M. (2002). The regulation of ammonium translocation in plant. *J. Exp. Bot.* 53 883–890. 10.1093/jexbot/53.370.883 11912231

[B51] Seki M. Umezawa T. Urano K. Shinozaki K. (2007). Regulatory metabolic networks in drought stress responses. *Curr. Opin. Plant Biol.* 10 296–302. 10.1016/j.pbi.2007.04.014 17468040

[B52] Shahnejat-Bushehri S. Nobmann B. Devi Allu A. Balazadeh S. (2016). JUB1 suppresses *Pseudomonas* syringae-induced defense responses through accumulation of DELLA proteins. *Plant Signal. Behav.* 11:e1181245. 10.1080/15592324.2016.1181245 27159137 PMC4973753

[B53] Shen X. Zhou Y. Duan L. Li Z. Eneji A. E. Li J. (2010). Silicon effects on photosynthesis and antioxidant parameters of soybean seedlings under drought and ultraviolet-B radiation. *J. Plant Physiol.* 167 1248–1252. 10.1016/j.jplph.2010.04.011 20713250

[B54] Shi Y. Zhang Y. Yao H. Wu J. Sun H. Gong H. (2014). Silicon improves seed germination and alleviates oxidative stress of bud seedlings in tomato under water deficit stress. *Plant Physiol. Biochem.* 78 27–36. 10.1016/j.plaphy.2014.02.009 24607576

[B55] Silvente S. Sobolev A. P. Lara M. (2012). Metabolite adjustments in drought tolerant and sensitive soybean genotypes in response to water stress. *PLoS One* 7:e38554. 10.1371/journal.pone.0038554 22685583 PMC3369847

[B56] Slocum R. D. (2005). Genes, enzymes and regulation of arginine biosynthesis in plants. *Plant Physiol. Biochem.* 43 729–745. 10.1016/j.plaphy.2005.06.007 16122935

[B57] Sui N. Zhou Z. Yu C. Liu R. Yang C. Zhang F. (2015). Yield and potassium use efficiency of cotton with wheat straw incorporation and potassium fertilization on soils with various conditions in the wheat-cotton rotation system. *Field Crops Res.* 172 132–144. 10.1016/j.fcr.2014.11.011

[B58] Tripathi P. Tripathi R. D. Singh R. P. Dwivedi S. Chakrabarty D. Trivedi P. K. (2013). Arsenite tolerance in rice (*Oryza sativa* L.) involves coordinated role of metabolic pathways of thiols and amino acids. *Environ. Sci. Pollut. Res.* 20 884–896. 10.1007/s11356-012-1205-1205 23054772

[B59] von Wiren N. Lauter F. R. Ninnemann O. Gillissen B. Walch-Liu P. Engels C. (2000). Differential regulation of three functional ammonium transporter genes by nitrogen in root hairs and by light in leaves of tomato. *Plant J.* 21 167–175. 10.1046/j.1365-313x.2000.00665.x 10743657

[B60] Waditee R. Bhuiyan M. N. H. Rai V. Aoki K. Tanaka Y. Hibino T. (2005). Genes for direct methylation of glycine provide high levels of glycinebetaine and abiotic-stress tolerance in *Synechococcus* and *Arabidopsis*. *Proc. Natl. Acad. Sci. U.S.A.* 102 1318–1323. 10.1073/pnas.0409017102 15665084 PMC547866

[B61] Waraich E. A. Ahmad R. Saifullah U. Ashraf M. Y. Ehsanullah (2011). Role of mineral nutrition in alleviation of drought stress in plants. *Aust. J. Crop Sci.* 5 764–777.

[B62] Wei J. Li C. Li Y. Jiang G. Cheng G. Zheng Y. (2013). Effects of external potassium (K) supply on drought tolerances of two contrasting winter wheat cultivars. *PLoS One* 8:e69737. 10.1371/journal.pone.0069737 23874992 PMC3707864

[B63] Wirtz M. Droux M. (2005). Synthesis of the sulfur amino acids: cysteine and methionine. *Photosynth. Res.* 86 345–362. 10.1007/s11120-005-8810-9 16307301

[B64] Witt S. Galicia L. Lisec J. Cairns J. Tiessen A. Araus J. L. (2012). Metabolic and phenotypic responses of greenhouse-grown maize hybrids to experimentally controlled drought stress. *Mol. Plant.* 5 401–417. 10.1093/mp/ssr102 22180467

[B65] Xia J. Sinelnikov I. V. Han B. Wishart D. S. (2015). Metaboanalyst 3.0-aking metabolomics more meaningful. *Nucleic Acids Res.* 43 W251–W257. 10.1093/nar/gkv380 25897128 PMC4489235

[B66] Yin L. Wang S. Li J. Tanaka K. Oka M. (2013). Application of silicon improves salt tolerance through ameliorating osmotic and ionic stresses in the seedling of *Sorghum bicolor*. *Acta Physiol. Plant.* 35 3099–3107. 10.1007/s11738-013-1343-5

[B67] Yin L. Wang S. Liu P. Wang W. Cao D. Deng X. (2014). Silicon-mediated changes in polyamine and 1-aminocyclopropane-1-carboxylic acid are involved in silicon-induced drought resistance in *Sorghum bicolor* L. *Plant Physiol. Biochem.* 80 268–277. 10.1016/j.plaphy.2014.04.014 24813726

[B68] Zarei A. Chiu G. Z. Yu G. Trobacher C. P. Shelp B. J. (2017). Salinity-regulated expression of genes involved in GABA metabolism and signaling. *Botany* 95 621–627. 10.1139/cjb-2016-0304

[B69] Zhao X. Wang W. Zhang F. Deng J. Li Z. Fu B. (2014). Comparative metabolite profiling of two rice genotypes with contrasting salt stress tolerance at the seedling stage. *PLoS One* 9:e108020. 10.1371/journal.pone.0108020 25265195 PMC4179258

