# Supplementary material for: Regulatory Role of Silicon in Mediating Differential Stress Tolerance Responses in Two Contrasting Tomato Genotypes Under Osmotic Stress
Source: Front Plant Sci. 2018 Oct 8;9:1475. doi: 10.3389/fpls.2018.01475 (PMC6187069; doi:10.3389/fpls.2018.01475)
Supplement: Supplementary file 1 [file Data_Sheet_1.PDF]

Supplementary Table S1. List of primers used for qRT-PCR.

GENE	ACCESSION NO.	FORWARD PRIMER	REVERSE PRIMER	AMPLICON SIZE (BP)
SlJUB1	XM_019213752	5’-AGTTCCACTTACACGAGGTCA-3’	5’-TGTTGGTTCCTGGTTTCATCC-3’	200
SlASS	NM_001329415	5’-GTCGTCAAAGTCCTCACAGTT-3’	5’-TAACCCTCATTGGAAGCCC-3’	121
SlASL	KJ807176	5’-TTGATGCCACTACACTCGCT-3’	5’-CGACAGACCTCCCGACTATG-3’	84
SlMS	NM_001320054	5’-TCAGTTTTCAGGGAGGGAGTT-3’	5’-GATGGTATTCTTGGAGAGTGGATG-3’	80
SlGAD1	AB359913	5’-CTGCGGGTTGTCATTAGAGAG-3’	5’-AAACGAGGAGGCTGTGTGT-3’	104
SlGAD3	AB359915	5’-CTCCACATTTGCTTCTCGCT-3’	5’-TTGGTATGCTGCCTCTTTGG-3’	88
SlP5CR	XM_004233202	5’-GCAACAACCGAAGATGGAGA-3’	5’-GTAAACCAGCAGCCACCC-3’	172
SlP5CS	U60267	5’-GTTCTGTTGAGTGATGTAGACGG-3’	5’-TCTTCCCACCCTGGACTTG-3’	132
SlGR	NM_001247314	5’-GGCTTCGGTTGCTGTTTGT-3’	5’-GTTCTGCCTCATAGTTCCATCC-3’	179
SlACTIN	NM_001308447	5’-CCAACAGAGAGAAGATGACCC-3’	5’-ACCAGAGTCCAACACAATACC-3’	128
SlEF1α	NM_001247106	5’-GATGATTCCCACCAAGCCCA-3’	5’-TGACAACACCGACAGCAACA-3’	107
SlCAC	SGN-U314153	5’-TCCTTGACTCGTCCGCCA-3’	5’-GCCACTCTTCTCCCACACC-3’	99
SlSAND	SGN-U316474	5’-CGTCGGAGTTTTCATCACCA-3’	5’-GTAACCCAGCACAGTAGAACA-3’	157

Supplementary Table S2. Influence of Si supply on concentrations of different metabolites and amino acids in the roots of two contrasting tomato genotypes under osmotic stress. Different letters denote significant differences according to Fischer's LSD test ($p < 0.05$; n = 4). Concentrations are expressed in ng mg^{-1} FW and mg g^{-1} DW (†). (nd = not detected). Abbreviations are as follows: PEP: phosphoenolpyruvate, GABA: gamma-aminobutyric acid.

Pathways	Metabolites	FERUM			LA0147		
		Control	OSC	OSC + Si	Control	OSC	OSC + Si
**Sugar Metabolism**	**Trehalose 6-P**	12.65 ± 2.60 [a]	14.48 ± 1.84 [a]	11.80 ± 3.18 [a]	11.41 ± 0.67 [a]	12.69 ± 1.16 [a]	11.08 ± 0.29 [a]
**Glycolysis**	**Glucose 6-P**	16.51 ± 0.55 [b]	19.13 ± 0.36 [ab]	18.30 ± 0.86 [b]	16.45 ± 0.79 [b]	21.96 ± 2.69 [a]	18.41 ± 0.92 [b]
**TCA cycle**	**Fumaric acid**	39.34 ± 0.99 [a]	50.92 ± 7.23 [a]	50.23 ± 5.07 [a]	40.14 ± 1.95 [a]	52.89 ± 4.90 [a]	43.72 ± 6.23 [a]
	**Malic acid**	36.89 ± 0.76 [a]	47.84 ± 6.63 [a]	47.42 ± 4.96 [a]	38.01 ± 1.75 [a]	47.94 ± 4.28 [a]	40.77 ± 6.07 [a]
**Organic acids**	**Gluconic acid**	4.98 ± 0.28 [a]	5.62 ± 0.33 [a]	5.85 ± 1.04 [a]	5.95 ± 0.21 [a]	5.46 ± 1.22 [a]	5.84 ± 0.14 [a]
	**Chlorogenic acid**	20.57 ± 4.74 [ab]	30.08 ± 4.10 [a]	21.11 ± 6.17 [ab]	17.70 ± 1.17 [b]	23.75 ± 0.11 [ab]	16.64 ± 0.44 [b]
**Amino acids**	**†Histidine**	nd	0.13 ± 0.02 [a]	0.16 ± 0.04 [a]	0.11 ± 0.00 [a]	0.13 ± 0.00 [a]	0.13 ± 0.01 [a]
	**†Serine**	0.48 ± 0.06 [a]	0.58 ± 0.08 [a]	0.71 ± 0.13 [a]	0.48 ± 0.04 [a]	0.56 ± 0.11 [a]	0.62 ± 0.04 [a]
	**†Glutamine**	4.70 ± 0.33 [a]	3.53 ± 0.03 [a]	5.32 ± 0.87 [a]	4.22 ± 0.30 [a]	4.82 ± 0.86 [a]	4.39 ± 0.86 [a]
	**†Glycine**	**0.06 ± 0.01 [c]**	**0.08 ± 0.01 [c]**	**0.19 ± 0.04 [ab]**	**0.06 ± 0.01 [c]**	**0.10 ±0.03 [bc]**	**0.23 ± 0.06 [a]**
	**†Arginine**	nd	0.13 ± 0.02 [a]	0.18 ± 0.03 [a]	nd	0.15 ± 0.03 [a]	0.14 ± 0.00 [a]
	**†Aspartate**	0.94 ± 0.04 [a]	0.66 ± 0.06 [ab]	0.71 ± 0.16 [ab]	0.92 ± 0.03 [a]	0.69 ± 0.06 [ab]	0.53 ± 0.20 [b]
	**Glutamate**	**37.12 ± 1.01 [cd]**	**49.04 ± 0.83 [bc]**	**62.22 ± 2.94 [a]**	**32.75 ± 3.85 [d]**	**55.91 ± 9.42 [ab]**	**46.83 ± 0.16 [bc]**
	**†Threonine**	0.30 ± 0.01 [b]	0.35 ± 0.03 [ab]	0.34 ± 0.01 [ab]	0.33 ± 0.02 [ab]	0.36 ± 0.02 [a]	0.34 ± 0.01 [ab]
	**†Alanine**	0.21 ± 0.07 [b]	0.47 ± 0.03 [ab]	1.31 ± 0.46 [a]	0.22 ± 0.03 [b]	0.81 ± 0.27 [ab]	1.39 ± 0.51 [a]
	**†Proline**	**0.10 ± 0.03 [b]**	**0.11 ± 0.00 [b]**	**0.19 ± 0.04 [a]**	**0.11 ± 0.01 [b]**	**0.13 ± 0.02 [ab]**	**0.11 ± 0.01 [ab]**
	**†Valine**	0.19 ± 0.01 [a]	0.32 ± 0.03 [a]	0.34 ± 0.11 [a]	0.20 ± 0.03 [a]	0.22 ± 0.03 [a]	0.22 ± 0.02 [a]
	**†Isoleucine**	0.12 ± 0.03 [a]	0.14 ± 0.01 [a]	0.13 ± 0.01 [a]	0.10 ± 0.01 [a]	0.15 ± 0.02 [a]	0.12 ± 0.01 [a]
	**†Leucine**	0.20 ± 0.02 [b]	0.26 ± 0.01 [ab]	0.30 ± 0.03 [a]	0.22 ± 0.01 [b]	0.31 ± 0.02 [a]	0.30 ± 0.03 [a]
	**†GABA**	**0.53 ± 0.04 [b]**	**1.00 ± 0.08 [ab]**	**1.80 ± 0.51 [a]**	**0.65 ± 0.11 [b]**	**1.20 ± 0.26 [ab]**	**1.72 ± 0.47 [a]**

Supplementary Table S3. Influence of Si supply on concentrations of different metabolites and amino acids in the leaves of two contrasting tomato genotypes under osmotic stress. Different letters denote significant differences according to Fischer's LSD test ($p < 0.05$; n = 4). Concentrations are expressed in ng mg^{-1} FW and mg g^{-1} DW (†). Abbreviations are as follows: PEP: phosphoenolpyruvate, GABA: gamma-aminobutyric acid.

Pathways	Metabolites	FERUM			LA0147		
		Control	OSC	OSC + Si	Control	OSC	OSC + Si
**Sugar Metabolism**	**Trehalose 6-P**	12.72 ± 0.88 ^{a}	13.33 ± 0.67 ^{a}	14.19 ± 1.03 ^{a}	12.15 ± 0.65 ^{a}	17.10 ± 3.29 ^{a}	17.52 ± 3.18 ^{a}
**Glycolysis**	**Glucose 6-P**	19.15 ± 0.80 ^{b}	21.93 ± 1.51 ^{ab}	20.92 ± 1.49 ^{ab}	24.30 ± 0.81 ^{ab}	24.61 ± 3.49 ^{a}	25.45 ± 1.45 ^{a}
	**PEP**	5.06 ± 0.74 ^{b}	6.75 ± 0.60 ^{a}	6.20 ± 0.30 ^{ab}	6.47 ± 0.28 ^{ab}	5.52 ± 0.03 ^{ab}	5.21 ± 0.42 ^{b}
**TCA cycle**	**Fumaric acid**	9.58 ± 0.09 ^{b}	14.32 ± 1.88 ^{a}	16.05 ± 1.89 ^{a}	8.85 ± 0.32 ^{b}	8.23 ± 0.42 ^{b}	10.72 ± 1.25 ^{b}
	**Malic acid**	**6.69 ± 0.37 ^{b}**	**9.29 ± 0.88 ^{b}**	**15.76 ± 4.42 ^{a}**	**5.80 ± 0.35 ^{b}**	**7.80 ± 1.15 ^{b}**	**6.67 ± 0.11 ^{b}**
	**Citric acid**	81.40 ± 5.99 ^{a}	87.25 ± 5.16 ^{a}	87.09 ± 6.05 ^{a}	78.53 ± 1.82 ^{a}	90.96 ± 8.77 ^{a}	79.97 ± 4.40 ^{a}
	**Isocitric acid**	64.18 ± 1.78 ^{c}	67.80 ± 1.35 ^{bc}	75.98 ± 5.07 ^{ab}	75.02 ± 1.10 ^{ab}	79.90 ± 4.66 ^{a}	79.94 ± 2.67 ^{a}
**Organic acids**	**Gluconic acid**	4.93 ± 0.11 ^{a}	6.32 ± 0.46 ^{a}	9.00 ± 1.73 ^{a}	6.10 ± 0.21 ^{a}	9.33 ± 3.16 ^{a}	6.61 ± 0.65 ^{a}
	**Chlorogenic acid**	43.80 ± 4.80 ^{a}	43.07 ± 4.86 ^{a}	40.76 ± 5.42 ^{a}	45.77 ± 1.80 ^{a}	48.31 ± 8.43 ^{a}	42.99 ± 5.52 ^{a}
**Amino acids**	**Histidine**	0.08 ± 0.00 ^{ab}	0.07 ± 0.00 ^{b}	0.13 ± 0.03 ^{ab}	0.06 ± 0.00 ^{b}	0.34 ± 0.21 ^{a}	0.12 ± 0.03 ^{ab}
	**†Serine**	**1.12 ± 0.01 ^{ab}**	**0.95 ± 0.08 ^{abc}**	**0.79 ± 0.20 ^{bc}**	**1.23 ± 0.12 ^{a}**	**0.68 ± 0.18 ^{c}**	**1.17 ± 0.11 ^{a}**
	**†Glutamine**	9.93 ± 1.09 ^{a}	6.51 ± 1.21 ^{ab}	5.53 ± 1.23 ^{b}	6.79 ± 0.66 ^{ab}	6.00 ± 1.68 ^{ab}	7.41 ± 1.56 ^{ab}
	**†Glycine**	**0.34 ± 0.02 ^{b}**	**0.30 ± 0.04 ^{b}**	**0.35 ± 0.19 ^{b}**	**0.61 ± 0.07 ^{ab}**	**0.26 ± 0.08 ^{b}**	**0.85 ± 0.21 ^{a}**
	**Arginine**	**0.15 ± 0.02 ^{c}**	**0.26 ± 0.08 ^{c}**	**0.18 ± 0.06 ^{c}**	**0.90 ± 0.00 ^{ab}**	**0.71 ± 0.26 ^{b}**	**1.07 ± 0.12 ^{a}**
	**†Aspartate**	1.65 ± 0.08 ^{a}	1.50 ± 0.03 ^{a}	1.63 ± 0.14 ^{a}	1.40 ± 0.06 ^{a}	1.29 ± 0.29 ^{a}	1.38 ± 0.02 ^{a}
	**†Glutamate**	34.09 ± 0.78 ^{ab}	35.34 ± 0.16 ^{ab}	36.55 ± 2.24 ^{a}	30.20 ± 0.45 ^{bc}	27.59 ± 3.67 ^{c}	33.45 ± 1.89 ^{abc}
	**†Threonine**	**0.73 ± 0.06 ^{abc}**	**0.60 ± 0.05 ^{abc}**	**0.51 ± 0.10 ^{c}**	**0.83 ± 0.07 ^{ab}**	**0.56 ± 0.18 ^{bc}**	**0.85 ± 0.09 ^{a}**
	**†Alanine**	**0.84 ± 0.02 ^{a}**	**0.59 ± 0.05 ^{b}**	**0.58 ± 0.10 ^{b}**	**0.87 ± 0.08 ^{a}**	**0.60 ± 0.12 ^{b}**	**0.99 ± 0.04 ^{a}**
	**†Proline**	**0.10 ± 0.00 ^{c}**	**0.14 ± 0.01 ^{bc}**	**0.24 ± 0.06 ^{a}**	**0.19 ± 0.03 ^{ab}**	**0.17 ± 0.01 ^{abc}**	**0.18 ± 0.02 ^{ab}**
	**†Valine**	0.13 ± 0.01 ^{ab}	0.10 ± 0.01 ^{c}	0.12 ± 0.01 ^{b}	0.12 ± 0.01 ^{ab}	0.12 ± 0.00 ^{abc}	0.15 ± 0.01 ^{a}
	**Isoleucine**	6.73 ± 0.09 ^{c}	7.67 ± 0.50 ^{c}	7.79 ± 0.83 ^{c}	10.84 ± 0.67 ^{ab}	8.78 ± 0.97 ^{bc}	12.68 ± 1.60 ^{a}
	**Leucine**	5.86 ± 0.78 ^{ab}	4.81 ± 0.72 ^{b}	6.53 ± 0.81 ^{ab}	5.72 ± 0.41 ^{ab}	6.74 ± 0.59 ^{ab}	7.81 ± 0.85 ^{a}
	**Methionine**	**3.21 ± 0.04 ^{b}**	**2.85 ± 0.18 ^{b}**	**1.98 ± 0.58 ^{b}**	**4.93 ± 0.25 ^{a}**	**2.61 ± 0.76 ^{b}**	**4.69 ± 0.27 ^{a}**
	**Phenylalanine**	10.60 ± 0.70 ^{bc}	6.16 ± 0.08 ^{d}	7.61 ± 1.20 ^{d}	13.70 ± 0.56 ^{a}	7.87 ± 0.90 ^{cd}	11.17 ± 1.44 ^{ab}
	**†GABA**	**0.83 ± 0.05 ^{b}**	**0.68 ± 0.07 ^{c}**	**1.02 ± 0.02 ^{a}**	**0.82 ± 0.04 ^{bc}**	**0.78 ± 0.44 ^{bc}**	**0.91 ± 0.03 ^{ab}**

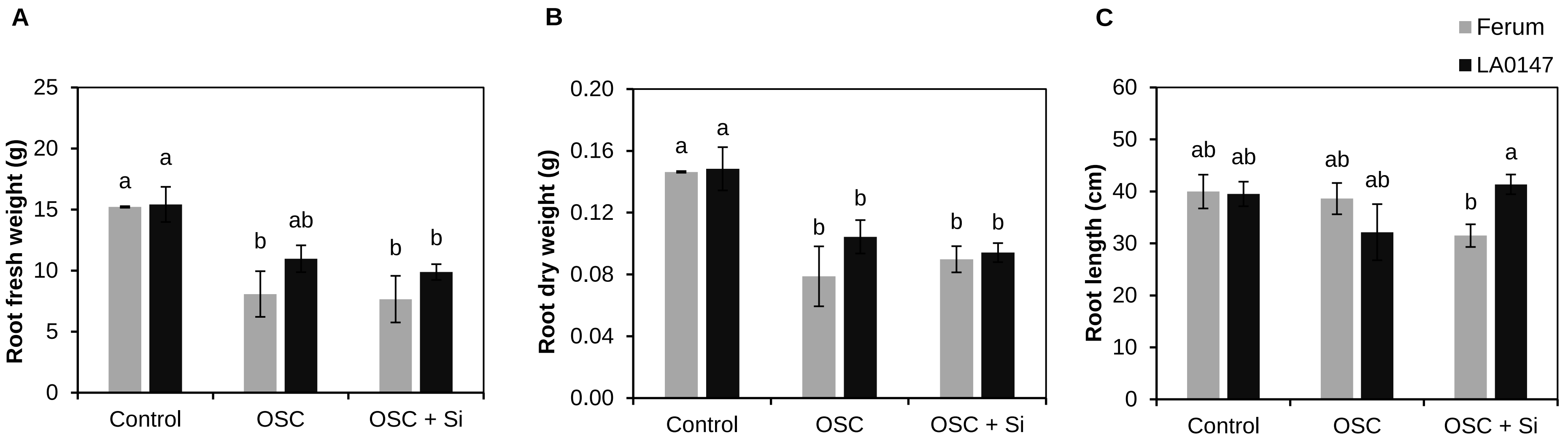

Supplementary Figure S1. Influence of Si supply on fresh weight, dry weight and length of roots in two contrasting tomato genotypes under osmotic stress. (A) root fresh weight, (B) root dry weight and (C) root length. Plants were grown in hydroponic culture and osmotic stress was simulated by applying polyethylene glycol (PEG 6000). Si was provided at 0.75 mM for pre-cultured plants and at 1.5 mM for osmotic stressed plants. Roots and fully expanded leaves from 21-day old plants were harvested 7 days after imposition of osmotic stress. Bars indicate means ± SE. Different letters denote significant differences according to Fischer's LSD test ($p < 0.05$; $n = 4$).

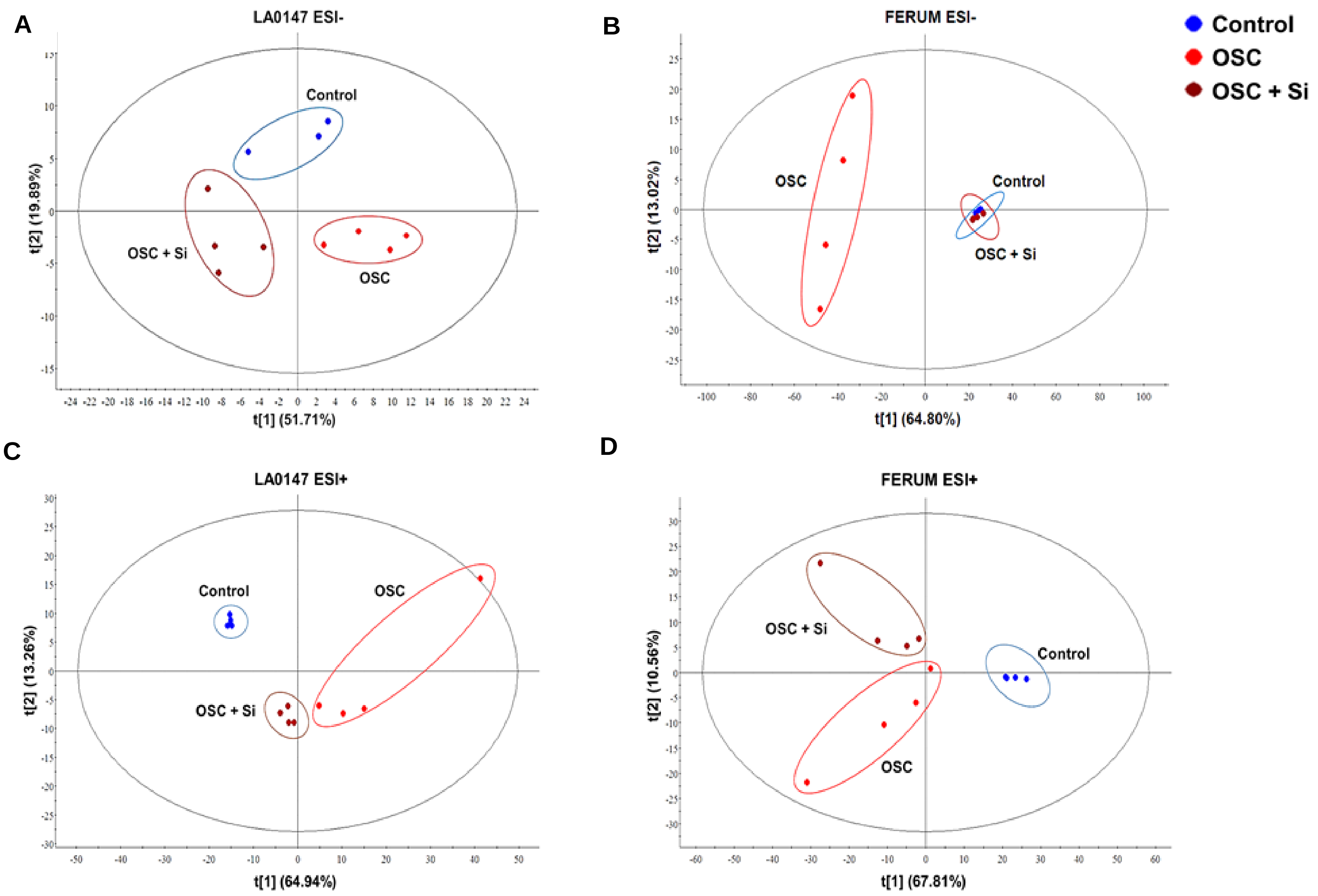

Supplementary Figure S2. Principal component analysis (PCA) based on identified metabolites in roots and leaves of two contrasting tomato genotypes. (A) PCA in roots of tolerant line LA0147, (B) PCA in root of sensitive line FERUM (C) PCA in leaves of tolerant line LA0147, (D) PCA in leaves of sensitive line FERUM.

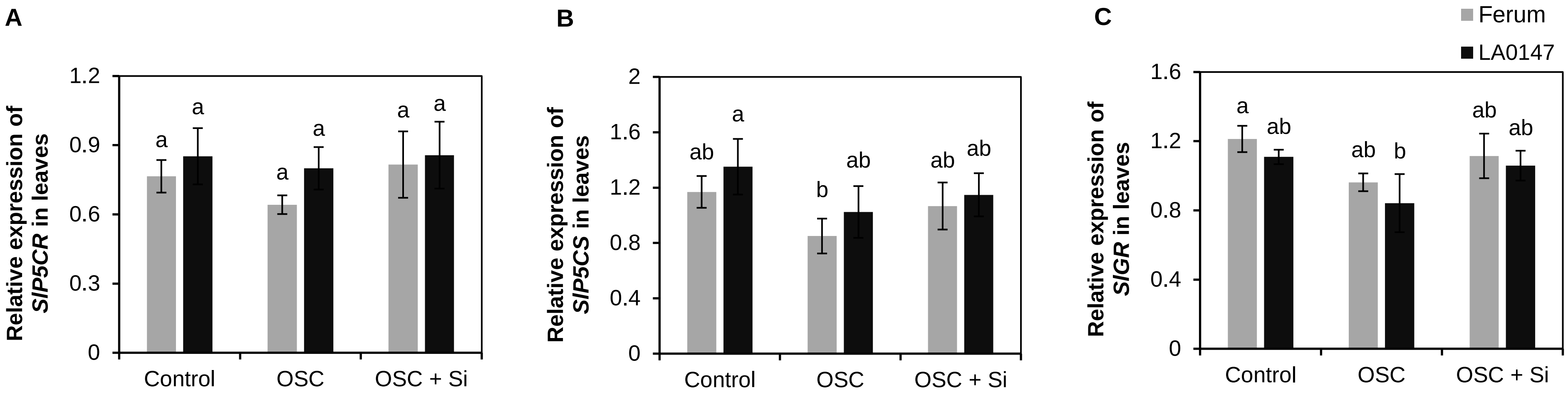

Supplementary Figure S3. Influence of Si supply on expression levels of the genes involved in proline and glutathione synthesis pathways in leaves of two contrasting tomato genotypes under osmotic stress. (A) relative expression of *SlP5CR*, (B) relative expression of *SlP5CS* and (C) relative expression of *SlGR*. Plants were grown in hydroponic culture and osmotic stress was simulated by applying polyethylene glycol (PEG 6000). Si was provided at 0.75 mM for pre-cultured plants and at 1.5 mM for osmotic stressed plants. Roots and fully expanded leaves from 21-day old plants were harvested 7 days after imposition of osmotic stress. Bars indicate means ± SE. Different letters denote significant differences according to Fischer's LSD test ($p < 0.05$; $n = 4$).
